# Expanded taxonomies of human memory

**DOI:** 10.3389/fcogn.2024.1505549

**Published:** 2025-01-15

**Authors:** Jason R. Finley

**Affiliations:** Department of Psychology, Southern Illinois University Edwardsville, Edwardsville, IL, United States

**Keywords:** memory, taxonomy, internal memory, external memory, collective memory, information storage, body memory

## Abstract

What is human memory? Evidence from cognitive psychology and neuroscience supports the view that human memory is composed of multiple subsystems. The influential “modal model” of the late 1960s proposed a sensory register, short-term store, and long-term store. Refinements and expansions to this taxonomy followed, including the construct of working memory, itself with several components, to replace earlier simpler ideas of short-term memory. Long-term memory appears to also consist of several subsystems, which can be broadly divided into explicit (declarative) vs. implicit (nondeclarative). Explicit long-term memory can be further subdivided into episodic vs. semantic, and implicit long-term memory includes subtypes such as procedural memory, priming, classical conditioning, and habituation. All of the above take place in the human brain, driven by neurons and the processes of long-term potentiation and depression. I previously proposed an expanded taxonomy that included external memory, which is information stored outside of an individual's brain, subdivided into social (information stored in other people) and technological (information stored in the human-made environment, either low-tech such as paper, or high-tech such as computers). In this manuscript, I propose even further expanded taxonomies of human memory, based on my view that memory is the transmission of information across time. The second expansion encompasses numerous biological systems beside the brain, including the immune system, genetics (DNA and epigenetics), and traces of the past stored elsewhere in the body (skin, hair, body modification, nails, bones and teeth, muscles and movement, voice, digestion and excretion, blood, reproductive systems, fat, lungs, and body-based numerical representation). The third expansion distinguishes between individual and collective memory (shared memory of a social group), revisits the other systems using the lens of collective memory, and adds natural external memory. Fruitful insights are possible from considering these expanded taxonomies using traditional ideas from cognitive psychology (e.g., encoding, storage, retrieval, forgetting). I explore numerous parallels, distinctions, and interplays.

## Introduction

Memory is the transmission of information across time. What then is human memory? Psychology has defined it as a complex system of encoding, storage, and retrieval accomplished by a human brain, and consisting of several subsystems. But should our conception of human memory be restricted to the information processes of an individual's brain? Or could it expand to include all aspects of what it means to be human? What might we gain from such an expanded framework?

In this manuscript, I present several expanded taxonomies of human memory that go beyond the current prevailing one. The first expansion comes from Finley et al. (2018),^1^ in which we proposed a taxonomy that includes external memory, which we defined as information stored outside of an individual's brain. The second and third expansions I first conceived of as part of a multi-disciplinary honors course I created at Fontbonne University, titled *Memory and the Human Experience*, with grant funding from the National Endowment for the Humanities (Finley, 2017). The second expansion adds several biological memory systems beside the brain, notably the immune system and genetics. The third expansion adds a distinction between individual and collective memory.

A construct as complex as human memory may be decomposed in a variety of ways (Barsalou, 1999; Brewer and Pani, 1983; Michaelian, 2011; Roediger et al., 1990, 2002, 2017; Schacter and Tulving, 1994). The dominant approach in cognitive psychology has been the division of human memory into multiple **memory systems**, an approach with roots in a structuralist tradition, as opposed to a functionalist tradition which would instead emphasize processes over systems (Jacoby, 1988; Roediger et al., 1990; Toth and Hunt, 1999). An entire edited volume exists on the tension between these theoretical approaches as they apply to just long-term memory (Foster and Jelicic, 1999).

Additionally, two different overall perspectives on memory have guided much of research, as described by Koriat and Goldsmith (1996), and roughly paralleling the distinction between structuralism and functionalism. The storehouse metaphor sees memory like a physical space containing discrete elements of information. This view has guided much laboratory research using simple stimuli, in the tradition of Ebbinghaus. The view emphasizes quantity (e.g., capacity space for storage), and the evaluation of memory performance based on **completeness**, using input-bound measures (e.g., out of the items presented, what quantity were remembered?). The correspondence metaphor sees memory as a representation that corresponds to varying extents with what truly happened in the past. This view has guided more naturalistic research, in the tradition of Bartlett. The view emphasizes quality, and the evaluation of memory performance based on **accuracy**, using output-bound measures (e.g., out of the material remembered, how much of it actually happened?). Although these two views are not mutually exclusive (e.g., Finley and Brewer, 2024), the storehouse view, like structuralism, is more germane to the subdivision of memory into multiple systems (sometimes even called stores), and to the use of taxonomies. In fact, adopting a correspondence or functionalist view can blur the boundaries between supposedly separate subsystems in a taxonomy, such as short-term/working memory vs. long-term memory in the brain. Some research suggests that while these two differ in capacity, their accuracy (quality, fidelity, or precision) is more similar than different (Brady et al., 2013; Xie et al., 2024), perhaps constrained by the same underlying structures in the medial temporal lobe (Xie et al., 2023). There is even a view of working memory as not a separate system per se, but more of a temporarily activated subset of long-term memory (Cowan, 1988, 2019; Foster et al., 2024). I think there is merit in both approaches (structuralist/storehouse vs. functionalist/correspondence). I view memory as an activity or process—the transmission of information across time—but one that occurs by way of storage, and that is the focus of this manuscript.

Supposing that we do adopt the structuralist/storehouse approach, several criteria have been proposed to distinguish between multiple memory systems. The two most common criteria are neurological and behavioral dissociations (Willingham and Goedert, 2001, Table 1). That is, when people with brain damage show impaired performance on one task but not another (neurological dissociation), and when the manipulation of a variable affects one measure of performance but not another (behavioral dissociation). Additional criteria include that each proposed memory system should have its own rules of operation, and operate on a certain kind of information (Schacter and Tulving, 1994; Sherry and Schacter, 1987). Although there are problems with using such criteria to distinguish multiple memory systems, and arguments against the overall approach (cf. everything cited so far), the concept of multiple systems nevertheless persists. Buckner (2007) argued that, despite the edges being blurry, the concept is “most useful when employed as a construct to integrate multiple levels of observation, both behavioral and neural.”

A useful way of organizing multiple memory systems is a **taxonomy**. A taxonomy is a scheme of classification, often hierarchical. Taxonomies are used to organize entities or concepts, and are found across scientific disciplines (e.g., taxonomies of organisms in biology, or of stars in astronomy). Importantly, there is often more than one defensible way to create a taxonomy; this is known as taxonomic pluralism. When it comes to taxonomies of memory, Willingham and Goedert (2001) argued that these are descriptive rather than explanatory, and so can inspire theory, although they cannot serve as theories themselves. In a similar vein, Tulving (2000) wrote about the importance of conceptual analysis of memory (“what” questions). It is in the same spirit that I present several expanded taxonomies of human memory, based on my conception of memory as the transmission of information across time.

My expansions should not offer much controversy regarding multiplicity of systems, as the new additions all depend on separate physical substrates from neurological memory (the brain) and from each other, or offer a different level or scope of analysis in the case of collective memory. Young (1965), as quoted by Buckner (2007), reflected that “The technique of pushing the analysis of the system as far as possible… seems to have brought increasing clarification.” By pushing the concept of human memory to the extreme, we may see illuminating parallels and differences across the different forms of memory. At the very least, an expanded perspective may be its own reward.

## Prevailing taxonomy: human memory in the brain

**Figure 1:**
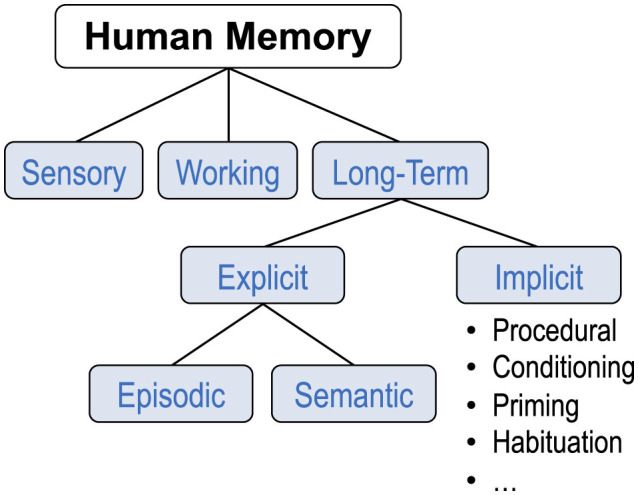
Prevailing taxonomy of human memory. Based on the original “modal model” by Atkinson and Shiffrin (1968) and later developments (e.g., Baddeley and Hitch, 1974; Anderson, 1976; Tulving, 1985; Squire, 2004).

**Figure 2:**
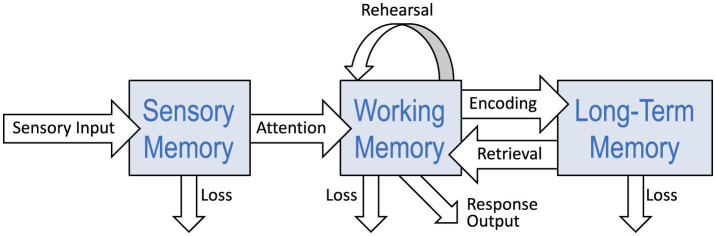
Box-and-arrows diagram of the three main subsystems in the prevailing taxonomy of human memory. Based on Atkinson and Shiffrin (1968), Shiffrin and Atkinson (1969).

Before presenting the expanded taxonomies, I will describe the prevailing one. Evidence from cognitive psychology and clinical neuroscience supports a current view of human memory as a complex faculty of the brain,^2^ consisting of a number of distinct yet interacting systems. Atkinson and Shiffrin's (1968; 2016) highly influential multi-store model proposed three major subsystems: sensory register, short-term store, and long-term store. Figure 1 depicts a taxonomy consisting of these three subsystems, but with an updated label for the second subsystem (working memory rather than short-term memory), and further divisions comprising long-term memory, all of which I will discuss shortly. Atkinson and Shiffrin (1968) also distinguished between the structures themselves and the control processes used to regulate storage and retrieval (mostly between the short-term and long-term stores). Their framework has come to be called the “modal model.”^3^ While being a taxonomy, the model is more often presented as a box-and-arrows flow diagram which includes visual depictions of control processes, as shown in Figure 2. In a retrospective essay, Atkinson and Shiffrin (2016) reflected on the importance of their work, while also hinting at the idea of external memory: “It is hard to imagine how understanding memory could not be important for the field and for humanity generally: Memory is what we are, and what defines us as individuals. Despite an ever-increasing reliance on external aids to memory (e.g., looking up forgotten material on the web), we rely on our memory for almost all decisions and interactions in our daily lives.”

### Sensory memory

The idea of sensory memory, also called sensory registers or buffers, is a very brief but complete and unprocessed (i.e., pre-categorical) copy of all environmental information entering through the senses, with different stores for the different senses. Energy from the world, such as light or sound, impinges upon human sensory neurons, such as rods and cones in the retina or hair cells in the inner ear. When stimulated, these specialized neurons send their electrochemical signals into the brain. The pattern of neural activation comprises the information. A subset of the information contained in sensory memory, as guided by attention, is passed to short-term/working memory for processing. The rest of the information decays rapidly and is lost (Loftus et al., 1992).

The most studied sense is vision, with visual sensory memory sometimes called iconic memory, and auditory sensory memory called echoic memory (Neisser, 1967). As demonstrated by Sperling (1960), the duration of sensory memory for vision is on the order of hundreds of milliseconds. Neural activity may persist in retinal ganglion cells, and in cortical neurons early in the visual processing stream (e.g., V1; Irwin and Thomas, 2008). Sensory memory can be thought of as persistence of sensory activity beyond the disappearance of a stimulus, yielding a modality-specific temporary preservation of the physical characteristics of a stimulus. A common example of persistence of vision is seeing a light trail behind a rapidly moving source of light such as a sparkler. You can also experience persistence of vision from simply waving your hand in front of your face.

Although sensory memory has been the subject of less research than short-term and long-term memory, there have nonetheless been developments and conundrums. For example, there has been debate on the extent to which different experimental procedures measure different underlying processes (Long, 1980; Irwin and Thomas, 2008). Coltheart (1980) argued that visual sensory memory consists of two separate stores for visual persistence and information persistence (see Irwin and Thomas, 2008 for a review). Cowan (1984) reviewed evidence showing two stores for auditory sensory memory. Cowan (1988) summarized: “…there do seem to be two distinct forms of memory for sensation in each modality: one lasting < 1 s, experienced as sensation, and a second lasting a number of seconds, experienced instead as a vivid recollection of the stimulus.” Because of the longer duration of some phenomena, it can be hard to tell where to draw the line between sensory memory and short-term memory. Roediger et al. (2002) summarized: “… attempts to categorize memories by their longevity are fraught with difficulties, as sharp boundaries do not exist. One type of memory usually blends into another.” There have been other conceptions too. Gross and Flombaum (2017) proposed viewing visual sensory memory as a kind of probabilistic representation. Ögmen and Herzog (2016) proposed that visual sensory memory consists of both a retinotopic reference frame and a motion-based reference frame, to account for normal viewing conditions when objects or the subject are in motion.

Some have questioned whether to call sensory memory a kind of memory at all. Baddeley (1999) wrote, of auditory sensory memory: “Although one would not term this a memory system in the usual sense, it certainly is a system for storing and retrieving information, and as such can legitimately be described as a very brief sensory memory system.” Cowan (2008) wrote: “It also may be that the brief sort of sensory memory is not truly a memory as such but, rather, a side effect of perceptual processing.” Sensory memory is perhaps more at home in discussions of perception than memory, and it is omitted from several authoritative reviews of the structure of human memory (e.g., De Brigard, 2023; Squire et al., 1993). In my view, however, sensory memory should be included, because it is indeed a transmission of information across time.

### Short-term/working memory

The second of the three major subsystems of human memory in the prevailing taxonomy is short-term memory, sometimes referred to as primary memory (James, 1890, Chapter XVI) and later reconceptualized as working memory. The idea of short-term memory is a brief and limited capacity store that contains information passed in from sensory memory (as filtered by attention) and/or information retrieved from long-term memory. The duration of short-term memory is ~15–20 s, as demonstrated notably by Brown (1958) and Peterson and Peterson (1959). After this duration, information is lost, due to decay and/or interference. However, rehearsal (a control process) can refresh this duration indefinitely. The limited capacity of short-term memory was estimated to be 7 ± 2 chunks of information by Miller (1956), later revised to 4 ± 1 chunks of information (Cowan, 2001, 2015).

Baddeley and Hitch (1974) effectively rebranded short-term memory as working memory, emphasizing that information in this store is actively manipulated to achieve complex tasks such as learning, reasoning, and comprehension (Baddeley, 2007, 2012). The idea is that working memory consists of several components: a phonological loop that holds auditory information, a visuo-spatial sketchpad that holds visual information, and a central executive that directs the processing of information in those stores (closely tied to attention; see Cowan, 2005). Further theoretical developments suggested additional components such as a multidimensional episodic buffer (Baddeley, 2000).

The limited capacity of working memory is the major bottleneck of human cognition. We can only hold so many things actively in mind at once. However, we can group discrete units of information into larger chunks by drawing on meaning from long-term memory. Working memory can be thought of as a mental workspace that contains our current conscious awareness (cf. global workspace hypothesis, Baars, 2002). Finally, working memory is also the origin of output (i.e., action) from the human information processing system. Behavior originates here as signals sent to motor neurons to initiate movement. Where is working memory in the brain? It is complicated, but imaging studies show activation in the prefrontal cortex, as well as cingulate and parietal cortices (Chai et al., 2018). This is consistent with evidence suggesting that both working memory and long-term memory share “a system of broad, partly overlapping and interconnected neocortical networks” (Fuster, 1998). Again, there is even a view of short-term/working memory as merely an activated subset of long-term memory, with attention guiding which parts of long-term memory are active, and with a limit in simultaneous activity that results in the capacity limit (Cowan, 1988, 2019; Foster et al., 2024), although such a view is challenged by data from functional neuroanatomy and dissociations in behavioral performance (Roediger et al., 1990).

### Long-term memory

The idea of long-term memory is a vast repository of information stored in the brain, from more than about 20 s ago. The capacity of human long-term memory appears to be essentially unlimited, or rather only limited by the amount of information one can encode in the human lifespan (Dudai, 1997; Landauer, 1986; Reber, 2010). Similarly, the duration of human long-term memory appears to be limited only by how long we can live (Bahrick et al., 1975). Information from working memory can be encoded into long-term memory, to varying extents depending on the encoding strategies used (e.g., elaborative vs. rote rehearsal). Information from long-term memory can be retrieved and brought back into working memory.

The information in long-term memory is stored as potentiated patterns of activation distributed across many neurons all over the cerebral cortex (Fuster, 1998; Jeong et al., 2015; Lashley, 1950). These patterns are created by the cellular processes of **long-term potentiation** and long-term depression (Kandel, 2001), which yield persistent changes in the strength of synaptic connections among neurons in vast constellations across the cortex.

Furthermore, there appear to be several different subsystems (or different formats of information) that comprise long-term memory, and this is where prior theories have most used taxonomies (e.g., Squire, 2004, Figure 1 which includes related brain areas). There has been much discussion on what exactly constitutes a memory system (Nyberg and Tulving, 1996; Roediger et al., 1990; Schacter and Tulving, 1994; Sherry and Schacter, 1987). Behavioral and clinical evidence (e.g., Cohen and Squire, 1980; Graf and Schacter, 1985; Robbins, 1996) suggest that long-term memory can be broadly divided into explicit (also called declarative) vs. implicit (also called nondeclarative; Anderson, 1976; Schacter, 1987; Squire et al., 1993). Explicit long-term memory involves conscious access to information from the past, and can generally be verbalized. Implicit long-term memory is reflected in changed performance as a result of prior experience and is not necessarily accompanied by conscious awareness, nor easily verbalized.

Further subdivisions are also supported by evidence. For explicit long-term memory, Tulving (1972, 1985) proposed a distinction between episodic (memory for past episodes of experience in a certain time and place) vs. semantic (memory for facts or general knowledge). Implicit long-term memory appears to consist of several subtypes, including procedural memory (i.e., memory for skills), priming, savings in relearning, classical conditioning, and perceptual learning.^4^ Although rarely mentioned in the cognitive psychology literature, the basic non-associative forms of learning—habituation and sensitization (Kandel, 2001)—should also fall under implicit long-term memory (Squire, 2007). It is less clear whether to consider everything under the umbrella of implicit memory as comprising separate subsystems (Bowers and Marsolek, 2003; Schacter, 1987), and there have been various theories. For example, Schacter (1990, 1992) proposed several perceptual representation subsystems to explain repetition priming effects.

It is also worth noting that even the explicit long-term memory categories of episodic and semantic are not necessarily exhaustive. For example, there are also merged representations of repeated events (repisodes; Neisser, 1981). And Rubin and Umanath (2015) proposed the theoretical construct of event memory as “the mental construction of a scene, real or imagined, for the past or the future.” Finally, it is not clear where, if anywhere, to place in the prevailing taxonomy the idea of *prospective memory*, which is remembering to do things in the future (McDaniel and Einstein, 2007).

## Expansion 1: internal vs. external memory

The human brain, in its breathtaking complexity, is capable of monumental achievements. However, it is not by sheer cognitive power alone that humans have come to dominate and even leave our home planet. For we are a tool using species. We have always sought to alter the environment and craft tools to extend the biological capabilities of our bodies. Hammers to crush, knives to cut, fire to warm, clothing to protect, vessels to contain, pigments to paint, clay and stylus to write. In fact, we can argue that our very cognition extends into the tools we use, a perspective sometimes summarized as 4E: cognition as embodied, embedded, enacted, and extended (Heras-Escribano and Lobo, 2020; Wilson, 2002).

One of humanity's greatest and most uplifting achievements is language, which grants us the ability to produce and comprehend a limitless combination of symbols. Our capacity for spoken language evolved at some point in the distant past. Much later, a mere 5,000 years ago, we invented written language, an externalization of spoken language, allowing us to transmit information across generations into the future without relying solely on oral tradition. Even before written language, archaeological evidence dating back at least 30,000 years shows that our ancestors used the environment to store information in the form of cave paintings (Aubert et al., 2014) and marked objects such as bones (d'Errico, 2001).

**Figure 3:**
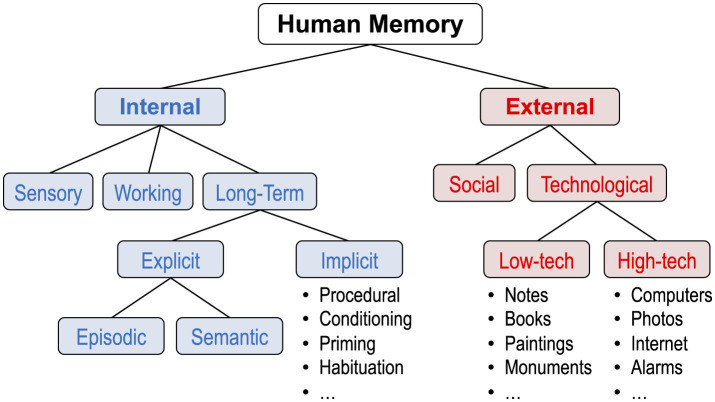
First expanded taxonomy of human memory. Changes from the prevailing taxonomy are as follows. There is now a distinction between internal memory (information stored in an individual's brain) and external memory (information stored outside of an individual's brain). External memory is subdivided into social (information stored in other people) and technological (information stored in the inanimate environment), with the latter including low-tech (not requiring a power source) and high-tech (requiring a power source). Adapted from Finley et al. (2018) Figure 1.1.

This all brings us to the idea of **external memory**, which is information stored outside of an individual human's brain. In our 2018 book, Finley et al. contrasted external memory with internal memory, information stored inside an individual human's brain (Finley et al., 2018).^5^ We proposed a taxonomy of human memory that included both internal and external memory, as shown in Figure 3.

We distinguished between several forms of external memory. We defined *social external memory* as information stored in other people, and technological external memory as information stored in the inanimate environment.^6^ For social external memory, Wegner et al. (1985) developed a theoretical framework called transactive memory, to study the distributed and collaborative nature of memory in human dyads (Huebner, 2016). Others have also written about collaborative memory (Harris et al., 2013, 2014; Rajaram and Maswood, 2018).

For *technological external memory* (see also Hertel, 1993; Nickerson, 2005; Schönpflug, 1986; Storm and Soares, 2024), we further divided it into low-tech, which does not require a power source to operate (e.g., paper), and high-tech, which does require a power source to operate (e.g., computer). Each individual has a *personal space of information* (Jones, 2007) that is available to us in external memory, whether it be low-tech, high-tech, or some combination. In 1945, Vannevar Bush conceived a then-theoretical recording and storage device, *memex*, that would provide an individual with “an enlarged intimate supplement to his memory” (Bush, 1945). With such abilities now in hand in the 21st century, the expanse of external memory surrounding us poses challenges in keeping (encoding), organizing, and finding (retrieval; Marshall, 2007). As Wegner (1987) said regarding expertise, “Knowing where things are to be found can be a more important consequence of education than merely knowing things.”

Note that our conception of external memory includes even information that has not yet been experienced by an individual, for example a movie they have not yet watched. In my view, what makes something a memory is that it is information from the past that may be recovered in the present. Among the many interesting findings from our comprehensive exploratory survey (Finley et al., 2018), we found that 81% of participants considered their external memories to be valuable, and 63% considered their external memories to be part of them.

### Justifying the distinctions

For internal memory, data from functional neuroanatomy and dissociations in behavioral performance (e.g., amnesic patients succeeding at one task but not another) are compelling reasons to propose multiple memory systems (e.g., Roediger et al., 1990). Sherry and Schacter (1987) discussed the plausibility, on evolutionary grounds, of multiple memory systems in the brain of a given species. They defined a memory system as “an interaction among acquisition, retention, and retrieval mechanisms that is characterized by certain rules of operation” and that two systems should be considered separate if they “are characterized by fundamentally different rules of operation.” Schacter and Tulving (1994) stated three criteria for a distinct memory system: “broad, category-based operations within a specifiable domain, a list of its properties that differentiate a given system from other systems, and relevant evidence in the form of converging task-comparison dissociations.” Those definitions were formed in order to consider the possibility of multiple systems within the same physical substrate, a brain. I think we can safely propose that memory achieved by a completely different physical substrate that is separate from the brain should be considered a separate system. Nevertheless, let us consider the extent to which the expanded distinctions in Figure 3 are justified in terms of properties and rules of operation.

### Internal memory vs. technological external memory

Finley et al. (2018, pp. 49–56, 157–159) detailed differences between internal memory and technological external memory based on survey data we gathered and our own reasoning. The main strengths of internal memory are rapid convenient access especially to frequently used information, rich vivid representations that include sensations and emotions, personally meaningful experiences/information, creativity, and security of private experiences/information. The main strengths of technological external memory are access to infrequently used information, accuracy (true representation of reality, where applicable), precision (exactness of details), longevity (long-lastingness), capacity (large amounts of information), fidelity (representations do not become distorted over time and reuse), and ease of social sharing (e.g., photos on social media). Additionally, internal memory is fundamentally associative and reconstructive in nature, while technological external memory is not. Both internal and technological external memory are susceptible to physical damage. Technological external memory can be duplicated (i.e., backed up) across multiple locations, but is susceptible to tampering by others. Furthermore, as derived from our exploratory survey and later replicated in two experiments (Finley and Naaz, 2023), people tend to strategically use these two forms of memory for different purposes, in ways that play to their strengths. Internal memory was favored for episodic and common procedural purposes, while technological external memory was favored for uncommon semantic, uncommon procedural, and far-term prospective purposes. The process of forgetting can be different between internal and external memory. Normal forgetting for internal memory may mean that the information still exists but is simply not retrievable at the moment (e.g., due to interference). For external memory, the information is more vulnerable to permanent and total loss (e.g., if a book is burned or a hard drive crashes, analogous to a stroke in the brain); this also means that intentional forgetting (erasure) is easier for external memory. Furthermore, although the decay theory of forgetting is not well supported for internal memory (McGeoch, 1932), decay is certainly possible in external memory (e.g., text on old tombstones becomes unreadable over time). Both internal and external memory are capable of storing information across different modalities, such as visual and auditory. That said, external memory is underdeveloped for some sensory modalities such as the chemical senses taste and smell, which are technologically more challenging to record and reproduce. Note how concepts from internal memory (e.g., episodic vs. semantic, forgetting) can be usefully applied to external memory.

### Social vs. technological external memory

What about the distinction between social and technological external memory? There have been some attempts to extend the transactive memory framework, originally developed for human dyads (Wegner et al., 1985), to technological external memory too (Wegner and Ward, 2013). However, Finley et al. (2018, pp. 75–77) argued that such an extension problematically obscures at least three qualitative differences between social and technological external memory. First, a person's technological external memory—their personal space of information—is generally fragmented across multiple mediums (e.g., photo albums, google searches, calendars, paper notes) and it is unclear how to characterize a subset of it as a memory partner like we would another human. Second, the retrieval processes of human and technological memory are fundamentally different. Human long-term memory reconstructs and infers information in ways vulnerable to distortion, whereas technological memory faithfully reproduces information. Third, technological external memory has no agency and little to no mental model of the human user's knowledge. Thus, it cannot contribute to self-regulation of a transactive memory system as a human member could. It cannot collaborate. However, recent advances in generative artificial intelligence (AI) may be changing this. I asked the AI ChatGPT-4 (July 30th, 2024) whether it has a mental model of the user, and it replied: “ChatGPT does not have a mental model of the user in the way humans do. However, it can remember context from the current conversation and, if enabled, can recall information from previous interactions. This helps in providing more relevant and personalized responses based on the user's preferences, interests, and prior queries. If there's anything specific you'd like me to remember about you to improve your experience, let me know!” Thus, the line has begun blurring between social and technological external memory (see also Fried, 2024; Hoskins, 2024), although meaningful differences persist.

### Low-tech vs. high-tech technological external memory

What about the distinction between levels of technological external memory? There are some important differences. High-tech external memory (especially at the level of computers, smartphones, and the Internet) has advantages such as searchability and easier duplication and transferability across devices and users. But it is also more complicated to use, and in some ways it is more fragile and ephemeral (Borgman, 2000, pp. 65–66, 202–205). High-tech external memory is more vulnerable to hardware failure, to hacking and viruses, to file corruption, and to obsolescence of file format, software, or hardware (e.g., floppy disks). For example, the BBC Domesday Project in 1986 compiled cultural information to be preserved for posterity on laserdisc technology, but this record was nearly unrecoverable a mere 16 years later due to obsolescence of software and hardware, not to mention copyright issues (Darlington et al., 2003). Meanwhile, the original paper Domesday Book, a census of England completed in the year 1086, has been carefully preserved for over 900 years and requires no special equipment to read. Low-tech external memory may be less flexible, but it is also less fragile and easier to use. It also includes using the spatial environment by placing objects to act as memory cues (McNamara, 2003; Winograd and Soloway, 1986). In short, there are both structural and functional differences in low-tech vs. high-tech external memory.

In terms of usage, Finley et al. (2018, pp. 64–66) found correlations such that older adults were more likely than younger adults to use low-tech external memory (e.g., shopping lists, bookmarks, notes on paper, and placing objects in specific places). In addition to level of technology, other dimensions of technological external memory worth exploring include: purpose of use (episodic, semantic, procedural, and prospective), encoding vs. retrieval processes, timescale, role relative to internal memory, and authorship and intended audience (Finley et al., 2018, pp. 157–165).

### Memory symbiosis framework

**Figure 4:**
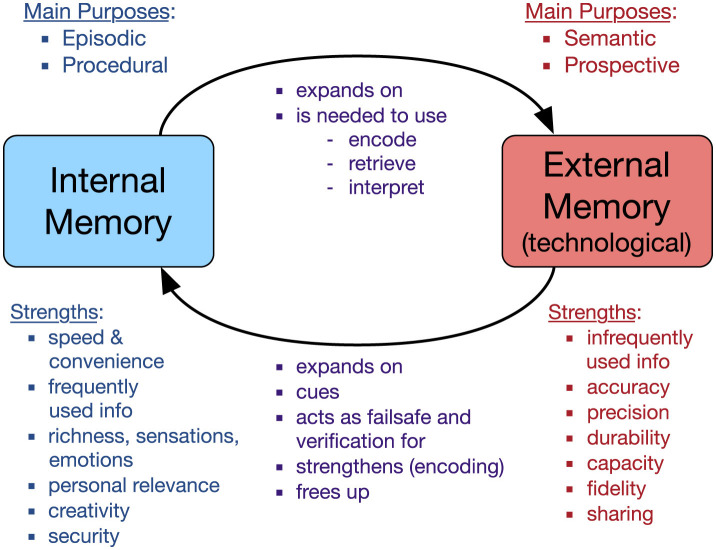
Memory symbiosis framework. Theoretical framework of the interplay between internal and external memory. Reprinted with permission from Finley et al. (2018). Version 1.0. Copyright Springer.

By separating internal and external memory into distinct components of the same overall system, reviewing several bodies of related literature, and asking participants about their attitudes, beliefs, behaviors, and experiences, we were able to explore the interplay between internal memory and technological external memory, yielding our Memory Symbiosis Framework, as shown in Figure 4.

We proposed that the two forms of memory, internal and external, are complementary and interdependent. In addition to listing their main purposes and strengths, the framework specifies ways in which they are related to each other (we give specific examples in Finley et al., 2018, Table 4.2). Internal memory can expand on technological external memory by providing rich recollections of original experience. Internal memory is also necessary to make use of technological external memory, to encode information externally in the first place, to remember that the information exists, to attempt to retrieve it when it is needed, to remember the location and procedure for retrieval, and to make sense of the information once retrieved. Without an accurate internal mental model of the location, structure, contents, and operational features of technological external memory, it may be inaccessible (Schönpflug, 1986).

In contrast, technological external memory is not needed to use internal memory. However, it is related to internal memory in several ways. Technological external memory can expand on internal memory with more detailed information than the gists of internal memory. It can cue (trigger) internal memory that would otherwise lie dormant (e.g., a photo reminding you of a holiday; see also Heersmink, 2023, “evocative objects”). It can serve as a fail-safe for when internal memory falters, and it can verify information retrieved from internal memory. The very act of encoding to technological external memory (e.g., taking notes) can strengthen internal memory (Jansen et al., 2017). Finally, offloading information to technological external memory can free up internal memory to be used for other tasks (Finley et al., 2018, Table 4.3).

To summarize, in the early 21st century, external memory appears to be augmenting internal memory for episodic purposes, and supplanting internal memory for semantic and prospective purposes. Since the invention of written language, the interplay between internal and external memory has shifted with the emergence of every new technology, and will undoubtedly continue to do so. For other authors' takes on this interplay, see Fawns (2011, 2020), Smart et al. (2017), Marsh and Rajaram (2019), Storm and Soares (2024), and an edited volume by Wang and Hoskins (2024).

## Expansion 2: memory elsewhere in the human body

**Figure 5:**
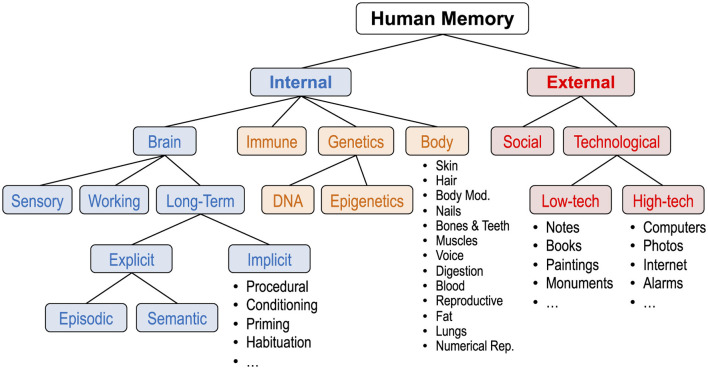
Second expanded taxonomy of human memory. Changes from the first expansion are as follows. External memory is now defined as information outside of an individual, and internal memory is now defined as information inside an individual (not specific to the brain). Internal memory is now subdivided into brain, immune, genetics (DNA and epigenetics), and body (including numerous systems).

The neurons of the brain are clearly the most powerful and intimate storehouse of human memory. That is the memory that psychologists study. But can we conceive of memory elsewhere in the human body? Yes. The past marks us, and we carry it with us in several ways. In this section, I expand the taxonomy to include memory in human biology outside of the brain, as shown in Figure 5.

### A word on “body memory”

Sometimes the term “body memory” or “somatic memory” is used to describe “a sensory recollection of trauma in the form of pain, arousal, tension, or discomfort, usually unaccompanied by words or images” (American Psychological Association, n.d.-a). In people with PTSD such recollections may “recur spontaneously or as a result of an environmental cue and typically revive unpleasant physiological phenomena” (Cohen, 1996, p. 528). It is important to note that such memories, although experienced as bodily sensations when retrieved, are *not actually stored in bodily tissue outside of the brain*. These kinds of memories, often retrieved involuntarily, could be considered episodic explicit long-term memories or procedural implicit long-term memories, depending on whether they are accompanied by conscious awareness that they are from a past episode. They are stored in the cerebral cortex just like all other neurological memories.

However, there is also a pseudoscientific idea of “body memory” as a complete record of past experience stored directly in bodily tissue independent of the brain, perhaps via some “cellular memory” at the level of individual cells (Smith, 1993). There is no evidence that this is possible, but the idea has been promoted by popular books about sexual abuse and trauma, along with the unfounded belief that unexplained bodily sensations can be evidence of repressed traumatic memories. For example: “the body remembers what the mind chooses to forget” (Bass and Davis, 1994, p. 83), “the body stores the memories of incest” (Blume, 1990, p. 279), “traumatic memory as it is stored in the brain and held in the body” (Levine, 2015, Introduction), and “memory of trauma is encoded in the viscera” (van der Kolk, 2014, Chapter 5 of the best-selling book, *The Body Keeps the Score*).

Unrelated to the above, there are several other uses of the term “body memory,” and these are not pseudoscience. Riva (2018) wrote about mental representation of one's own body as guided by proprioception, interoception, and vestibular senses, and influenced by social factors. Tewes and Fuchs (2018) introduced a special issue exploring the idea of body memory as habits and embodied practices in the context of cultural norms and styles of expression.

In my writing here about memory in the human body, I am not talking about any of the above uses of “body memory,” but rather am talking about verifiable ways that information from the past can indeed be stored in and retrieved from the human body outside the brain. I am not the first to do so. For example, Roediger (2003) said, “there is a sense that overt physical changes in an individual can signal their past experiences.” He considered memory in the body and gave examples of muscles, the immune system, and the female reproductive system, the latter two of which exhibit priming, which is a primary characteristic of implicit memory. However, he ultimately cautioned against considering these as memory systems, saying:

These systems evolved to serve some other biological purpose and they have a dash of memory tossed in to help the system profit from experience. Hence, calling them memory systems stretches the term too far. (p. 14)

My goal here is to stretch the term too far.

### The immune system

The immune system is stupefyingly complex, second only to the nervous system. It very clearly has its own kind of memory, separate from that of the brain (Ahmed and Gray, 1996; Roediger, 2003). Immunity is resistance to disease, which is achieved in part by the immune system's memory. The immune response has two major parts, innate and adaptive; immunological memory exists in the adaptive part. Here I sketch a simplified description of the immune response.

- Innate immune response:
  ○ First, a pathogen such as a bacterium or virus enters the body. It has certain molecular patterns on its surface. These are detected by pathogen recognition receptors, which are specialized protein structures on white blood cells (of which there are several types).
  ○ Second, inflammation is triggered, which increases blood flow and draws more white blood cells to the site of infection where they can neutralize the pathogens. For example, macrophage white blood cells kill pathogens by engulfing them.
- Adaptive immune response:
  ○ *Primary response*: Several days later, different white blood cells arrive: naive T and B cells. These become activated when they encounter antigens, which are molecules resulting from the actions of the pathogens. Once activated, some of the T cells can then kill infected cells that have the antigen. Activated B cells can produce antibodies, which are specialized protein molecules that neutralize the antigens.
  ○ **Encoding** happens, whereby some of the T and B cells become **memory cells**, which store the pattern of the antigen and the antibodies. These will then circulate in the blood and accumulate in places such as lymph nodes and the spleen, where they can be dormant for decades, analogous to long-term memory in the brain. However, this immunological encoding process can take days to weeks, much slower than encoding in the brain (Rasch and Born, 2013).
  ○ *Secondary response*: If the same or similar pathogen infects the body again at a later date, producing the same antigen, then **retrieval** happens when free-floating memory T and B cells recognize the antigen and mount an immune response that is stronger (producing more antibodies) and faster (a few days vs. a week or more) than the original primary immune response. In this way, “The immune system can remember, sometimes for a lifetime, the identity of a pathogen” (Ahmed and Gray, 1996).

Thus, immune memory is marked by faster and stronger reactions to previously encountered antigens, as mediated by the memory T and B white blood cells. This strengthened response is a kind of **priming** (or perhaps savings in relearning). **Natural killer cells** are another kind of white blood cell. Their role is to identify and kill infected or tumor cells, but they must first **learn** to discriminate between healthy cells that carry “self” markers, and unhealthy cells that do not. Thus, they too exhibit memory. There are several theories as to how natural killer cells are “educated” (Boudreau and Hsu, 2018). There is even evidence that these cells exhibit immunological memory similar to the T and B cells, for example by adapting to respond more effectively to previously encountered viruses (Cerwenka and Lanier, 2016).

**Natural immunity** is achieved by having memory cells from a prior infection. **Artificial immunity** can be achieved by vaccination, which is another method of encoding to teach the immune system (Sallusto et al., 2010). With traditional vaccines, a patient is injected with a weakened or neutralized form of a pathogen (e.g., Measles virus), and the immune system responds by producing appropriate antibodies and memory cells to lay in wait should the real pathogen ever be encountered. Some newer vaccines use mRNA, rather than neutralized pathogens, to stimulate the creation of antibodies. Analogous to the well-known **spacing effect** in neurological memory (Cepeda et al., 2008; Dempster, 1988), artificial immunity is often best achieved by administering multiple doses of a vaccine spaced apart in time. The delay between doses is based on time needed for encoding (i.e., development of memory T and B cells; Centers for Disease Control Prevention, 2023; Sallusto et al., 2010).

Immunotherapy is an increasingly promising class of treatments for cancer that artificially stimulates the immune system to better attack tumor cells (American Cancer Society, n.d.; Waldman et al., 2020). For example, chimeric antigen receptor T-cell therapy involves adding a gene to a patient's T cells in order to teach them how to attack tumor cells. Cancer vaccines are another example of immunotherapy, and include two categories: prophylactically inoculating against cancer-causing viruses (e.g., human papillomavirus), and therapeutically introducing a vaccine designed to train the immune system to recognize neoantigens produced by tumor cells. Patients who survived melanoma thanks to immunotherapy were found to maintain memory T cells calibrated against the cancer up to 9 years later (Han et al., 2021). Thus, scientific understanding of immunological memory enables us to harness that system for preventing and treating cancer.

Allergies are a vexing but interesting phenomenon of the immune system. White blood cells of the innate immune system encounter a generally harmless substance called an allergen (e.g., pollen), but falsely identify it as a pathogen. They then communicate to B cells which produce antibodies (immunoglobulin E) to fight the allergen, and these antibodies attach to mast cells. Some of the B cells turn into memory B cells, encoding the pattern of the allergen and the antibody. The next time the allergen is encountered, it will bind to the antibodies on the mast cells, which then release histamine, triggering the symptoms of an allergic response. The memory B cells mobilize to release even more antibodies, faster than the first time (Pfützner et al., 2023). Thus the allergic response is amplified. In this case, memory is maladaptive, analogous to misidentification (false recognition) in an eyewitness memory scenario.

Autoimmune diseases are also somewhat analogous to **false memory** in the brain, when people remember something that did not happen (e.g., misidentifying an innocent suspect in a lineup as the perpetrator of a crime). Autoimmune diseases occur when the immune system falsely recognizes healthy tissue as a pathogen and mobilizes an immune response to fight it (Davidson and Diamond, 2001).

Five key dimensions of immunological memory were identified by Pradeu and Du Pasquier (2018): strength of a second response to a pathogen relative to the first response, duration of the second response, speed of the second response, specificity of the second response, and extinction. Note that some of these are the same terms used in studies of classical and operant conditioning, which rely on neurological memory. In another analogy, both immunological and neurological memory appear to undergo system **consolidation** during sleep (Rasch and Born, 2013, pp. 733–736; Westermann et al., 2015). Unlike neurological memory, the capacity of long-term immunological memory appears to be limited, in that there is a maximum number of memory T and B cells (Sallusto et al., 2010).^7^ This makes the issue of **interference** important. If there is only limited capacity for memory T and B cells, then there may be competition among them (Freitas and Rocha, 2000).

To what extent can immunological memory and neurological memory operate simultaneously? When the immune system is actively fighting an infection, cognition appears to be generally impaired in terms of alertness and reaction time (Smith, 1990; Smith et al., 1998). A large-scale study by Katan et al. (2013) found that exposure to common bacterial and viral infections was negatively correlated with simple cognitive performance (e.g., the Mini-Mental State Examination). What about memory performance specifically? Laboratory research has found that, compared to healthy baseline, participants with an upper respiratory tract infection (e.g., cold, flu) show impaired *speed* in some tasks measuring working memory, episodic long-term memory, and semantic long-term memory, but not impaired memory accuracy (Bucks et al., 2008; Smith, 2012; Smith et al., 1990). However, one study (Capuron et al., 1999) did find that when compared to healthy participants, participants with flu-like symptoms, whether presenting with a fever or not, showed impaired accuracy scores on a variety of memory tasks including prospective memory, general knowledge, picture recognition, recall of a news article, and recall of a new spatial route.

What is the mechanism by which the immune system influences cognition in the brain? Research suggests the key is in cytokines (Dantzer, 2001), which are signaling proteins that coordinate the overall immune response. Some cytokines (e.g., interleukin-15) appear to be involved in making memory T cells (Schluns and Lefrançois, 2003). Another cytokine, interleukin-1β, promotes the body's inflammation response to infection. Rats injected with such cytokines into the brain show impaired learning (e.g., in a water maze task, Oitzl et al., 1993). The brain, perhaps in response to a combination of cytokines released throughout the body, produces its own interleukin-1β, which appears to disrupt hippocampal plasticity by interfering with long-term potentiation, thus impairing memory consolidation (Thomson, 2004). Thus, the operation of immunological memory, when actively fighting infection, can interfere with the regular operation of neurological memory. Ongoing research is investigating potential memory and other cognitive impairments following immunotherapy cancer treatment (Joly et al., 2020).

### Genetics

#### DNA

Deoxyribonucleic acid (DNA) is a complex molecule carrying the genetic code of an individual organism, which includes instructions for making proteins and ribosomes. Human DNA is packaged into 23 pairs of chromosomes stored in the nuclei of cells. The human genome, which is the entirety of an individual's genetic material (i.e., DNA), comprises ~3.2 billion base pairs of nucleotide molecules (adenine with thymine, or guanine with cytosine). This is a staggering amount of information, comprising the biological blueprint for the entire body.

Importantly, DNA can replicate itself. This is what makes it a kind of molecular memory, transmitting information across time. When a cell splits in the process of mitosis, both resulting cells retain a complete copy of the DNA. This is a useful kind of redundant memory storage, and preserves the information contained in the DNA across the lifetime of the individual. Ensuring the accuracy of the genetic memory requires constant self-repair, because DNA is frequently damaged by errors in replication or by exposure to mutagens (e.g., chemicals or ionizing radiation). Such damage is called a mutation, and cells have several systems for repairing them (Lindahl and Wood, 1999). In this way, genetic memory is unlike neurological memory, which is generally imprecise, malleable, prone to change over time and usage (e.g., reconsolidation), and has no mechanism for ensuring integrity.

The processes of transcription and translation can be considered a kind of memory retrieval. Transcription copies a segment of genetic code from the DNA to a strand of messenger RNA, and translation decodes the mRNA to make proteins essential for cellular function. What about encoding? Can DNA be altered to contain new information? Yes, with difficulty. Gene therapy is the process of changing an individual's DNA by, for example, inserting a new gene (i.e., a segment of DNA) to replace a defective gene. CRISPR is a method for such gene editing. Diseases such as hemophilia and lymphoma have been treated with gene therapy (Dunbar et al., 2018). This is analogous to, say, the misinformation effect or other methods of creating false memories in the brain, for therapeutic purposes. Finally, modern techniques of DNA profiling can be used to match DNA between a sample and a person (or database), or between multiple people. This kind of memory retrieval is useful for contexts such as genealogy, paternity testing, or criminal investigations (Glynn, 2022).

#### Epigenetics

Barring gene therapy or mutations, a person's DNA does not change across their whole life. However, *epigenetics* can change how genes in the DNA are expressed. Epigenetics is when factors such as normal development, environmental chemicals, drugs, aging, stress, or diet result in a process that does not alter the DNA itself, but alters how it is used by the cell. One example is when a methyl group molecule binds to a segment of DNA, acting to either repress or enhance the transcription of that gene. This is analogous to blocking or boosting a memory without altering it. Another example is modifying the shape of the histone molecule that DNA is usually wrapped around, which can prevent the unwinding necessary for that segment of DNA to be transcribed.

Whether on the DNA itself or the histones the DNA is wrapped around, epigenetic markers accrue over a lifetime and are persistent records of a past experience, and thus constitute a secondary form of memory riding on the DNA. In fact, perhaps we can think of epigenetics as a kind of *metamemory*. Furthermore, the epigenetic markers are passed on when cells divide in mitosis (Probst et al., 2009). And there is evidence that epigenetics plays a role in the process of long-term potentiation, which guides the structural changes in neurons that underlie memory in the brain (Bernstein, 2022; Fischer, 2014).

### The rest of the body

#### Skin

Skin is the largest organ of the human body, and the one in most contact with the environment. Our skin carries a wide range of information from the past, and thus constitutes a form of memory. As with bones and teeth, patterns of wear can indicate past activities. Calluses and corns are areas of thickened skin (lichenification) caused by repeated pressure or friction, often on the hands and feet. The particular pattern can be due to someone's occupations (Gradenwitz, 1907; Kanerva, 2000; Samitz, 1985). Ronchese (1948) provided a fascinating illustrated guide of various occupational marks on the skin. For example, string musicians may develop calluses on their fingertips (Önder et al., 1999), and people who work extensively with scissors (e.g., tailors, hairdressers) may develop calluses on the thumb and parts of one or two other fingers depending on how the tool is held (Vetrichevvel et al., 2008). Excessive computer mouse use can lead to a callus on the wrist (Goksugur and Cakici, 2006). Athletic activities such as dancing, running, basketball, or soccer can lead to calluses and other conditions on the feet (Bergfeld and Taylor, 1985). Foot calluses can also indicate time spent wearing poorly fitting footwear. Another common skin condition that reflects occupation is contact dermatitis, a rash caused by contact with an irritant or allergen. Different occupations yield different patterns of dermatitis across the body (Srinivas and Sethy, 2022; White, 2024). These kinds of mark patterns are akin to what Neisser (1981) called “repisodic” memory in the brain, because they are formed from an accumulation of many similar actions across episodes, rather than from one specific episode.

Scars show where skin was once injured and healed, whether from injury, illness, or surgery. They can even convey temporal information: younger scars are more likely to be red and bulge outward (i.e., hypertrophy; Kim et al., 2024; Lin and Lai, 2024). Different conditions yield different patterns of scars; for example, burn scars look different from acne scars. Stretch marks (striae) are scars that indicate prior growth spurts, for example due to puberty, pregnancy, body-building, or obesity. Dramatic weight loss can lead to excess skin, which may be surgically removed. There have even been cultures in which people make cuts on their own body to mark the number of enemies killed (Lagercrantz, 1973). Whatever their origin, scars can carry memory across many years.

Shorter-term skin memory can tell of injuries in the more recent past, as indicated by bruises, welts, minor cuts, scrapes, or abrasions. Scabs indicate ongoing healing from a recent injury. Insect bites leave red spots. Bruises change color with time, and typically disappear after around 2 weeks (Langlois and Gresham, 1991), although this can take longer with aging (National Institute on Aging, 2017). However, the variance in progression of coloration across people means that it is difficult to reliably estimate the age of a bruise, for example for forensic purposes in cases of child abuse, elder abuse, or domestic violence (Nash and Sheridan, 2009).

Skin hygiene also carries information. The presence of dirt shows that a person has recently gotten dirty, and/or has not recently bathed. Poor hygiene can be a warning sign of depression, dementia, abuse, or neglect (Stewart et al., 2022). Salt residue on skin can indicate recent physical exertion and sweating. Body odor can indicate recent exertion and/or lack of bathing, or could indicate health conditions. Application of soap, lotion, cream, deodorant, or perfume/cologne can result in lingering olfactory cues from the skin, typically lasting hours.

There are a variety of other skin conditions that reflect past experience. Dermatological exams involve questions about changes in skin; for example changes in the color, size, or shape of a mole can be warning signs of melanoma, a type of skin cancer (National Cancer Institute, 2022). Skin even hosts some immunological memory in the form of resident memory T cells, which can persist for years. These cells protect against pathogens but can also contribute to chronic inflammatory skin diseases such as psoriasis, which is an autoimmune disease (Chen and Shen, 2020; Pham et al., 2023). This is like a form of recurrent intrusive involuntary memory.

##### Skin pigmentation

The overall pigmentation (color) of skin carries information too. Pigmentation is a function of melanin, the amount of which is influenced by a complex genetic heritage and its interaction with the environment (Crawford et al., 2017; Martin et al., 2017). A person's pigmentation may darken due to recent tanning from sun exposure or tanning beds. Freckles also become more pronounced from sun exposure. The redness of a sunburn, followed by peeling skin, is clear evidence that someone spent too much time in direct sunlight without proper sun protection, and that this likely happened in the last 3–5 days (Johns Hopkins Medicine, n.d.). Other temporary changes in pigmentation (e.g., blushing, paleness, jaundice) carry information too, about current and recent health conditions or emotions. Dark baggy skin under the eyes can signal recent sleep deprivation.

##### Skin and aging

Skin holds information about the longer passage of time (i.e., aging; Pedersen et al., 2024). Intrinsic aging happens naturally, and the effects on skin include dryness, sagging, and fine wrinkling. The characteristics of skin also carry information about the kind of life one has lived. For example, extrinsic aging due to cumulative unprotected sun exposure (ultraviolet radiation), poor sleep, or a history of smoking tobacco can cause rough texture, change in pigmentation (e.g., age spots), and coarse wrinkling. Effects vary across skin pigmentations. It is possible to slow down the encoding of such signs of skin aging by avoiding unprotected UV exposure, avoiding smoking, and keeping skin moisturized. In some cases, such skin memories of aging can be overwritten (analogous to intentional forgetting) via cosmetic surgery or botox treatment. Dynamic wrinkles are those that appear on one's face when making different facial expressions. These can form into static wrinkles over years of repetition. For example, vertical lines between the eyebrows can indicate a history of frowning or scowling, and crow's feet wrinkles around the eyes can indicate a history of smiling or squinting. These are long-term cumulative marks of emotional expressions, akin to repisodic memory.

##### Fingerprints

Human fingertips feature complex, durable, and idiosyncratic patterns of friction ridges that form during fetal development and last a lifetime. The impression these patterns leave on surfaces is called a fingerprint, and is widely used as a biometric identifier for security systems and criminal investigations. However, there has been debate about the quality of the research behind the use of fingerprint identification for forensic purposes (Champod, 2015). We can think of one's fingertip patterns as semantic memory in the skin, because it is unchanging and not tied to any particular context. In contrast, scars are episodic memory in the skin, representing something that happened at a particular time and place in the past, and patterns of wear such as calluses and dynamic wrinkles are repisodic memory in the skin.

#### Hair

Humans are mammals, and thus we have an abundance of hair, which is a protein filament that grows from our skin. Hair carries information about the past. The distribution of hair across one's body, and the characteristics of the hair (e.g., thickness, curliness, volume, and color) reflect one's genetics (Shimomura and Christiano, 2010). Hair changes as we age, so its current condition conveys information about development. For example, the presence of pubic and underarm hair, and facial hair especially in males, is a record that an individual has undergone the secondary sexual changes of puberty. Hair can also indicate adult age, although not always precisely. With aging, hair tends to turn gray or white, become thinner, and/or disappear (i.e., balding). The age at which such changes happen varies across individuals and ethnicities (Maymone et al., 2021).

Hair loss (i.e., alopecia) can be considered analogous to forgetting, the loss of information that was once there. However, unlike with forgetting in the brain, the absence of hair passively visually conveys information. That is, the absence *is* information. Hair loss or thinning can happen due to aging (e.g., pattern baldness) as influenced by genetics and androgenic hormones (e.g., during menopause for women). There are other causes too, such as chemotherapy (cancer treatment), traction alopecia (due to tightly pulled hair styles), trichotillomania (due to compulsive hair pulling), and autoimmune disease (Harries et al., 2024).

Humans put great effort into removing, maintaining, cutting, trimming, coloring, and/or styling their hair, particularly on the head. Women in some cultures remove their body hair (depilation; Tiggemann and Hodgson, 2008). Men in some cultures cultivate and style their facial hair (Peterkin, 2001). One's current hair condition is a reflection of the past. If its length is very short, it was cut recently. If its length is very long, it has been growing for a long time (potentially years). Conditions such as split ends are a record of damage due to dryness. This can reflect that the individual has not recently had time or resources to care for their hair. Unkempt hair can also be an indicator of mental disorders such as depression (Beach et al., 2021). Hair can also be damaged by certain styling actions and products. For example, chemical hair relaxers used to straighten curly hair can strip the cuticle (outer layer of the hair shaft), weakening the hair. Tangled and tousled hair can indicate recent activities, such as sleep (“bed head”), sex, exercise, exposure to wind, or wearing certain articles of clothing (e.g., “hat head”).

Humans use many hair care products in order to style, color, and moisturize their hair. When hair has a color beside its innate color, this is a record of recent hair dying or bleaching, which humans have practiced for thousands of years (Stenn, 2016, pp. 131–133). As the hair grows, the roots reappear with the innate color, and the length of this section indicates the amount of time since the dying. Hair color can also change with exposure to the sun. Styled hair that holds a shape can be considered a kind of material memory (cf. Lexcellent, 2019)—the shape reflects the actions, temperatures, and products applied to it. Hair styles can convey cultural meaning too, which I will return to under Collective Memory.

Even after being removed from the body, human hair can function as external memory. For example, locks of hair may act as tokens for romantic exchange or keepsakes in remembrance of someone deceased (Stenn, 2016, pp. 111–117). The hair follicles themselves, because they are each fed by a blood vessel, absorb any drugs or heavy metals in a person's system and thus can be used for drug testing by employers, courts, law enforcement, or healthcare professionals. Thus hair displays a kind of chemical memory lasting up to months (e.g., 3 months for a 3 cm section; Gryczynski et al., 2014; Kintz et al., 2006).

#### Body modification

Body modification is deliberate alteration of the body for non-medical reasons. Common examples include tattoos, piercings, and implants. Other forms of body modification include tongue splitting, genital modification (e.g., female genital mutilation, male circumcision), foot binding, corsetry, ear stretching, neck or lip elongation, branding, scarification, and tooth filing (DeMello, 2007; Ward, 2022). Cosmetic surgery and tanning may be considered body modification too.

Such modifications may be made for personal and/or cultural reasons. For example, modifications may mark group membership or social status, alter the body to conform to cultural beauty standards (Wegenstein, 2012), or convey personal information about one's identity, experiences, or accomplishments (Bradley University, n.d.). Ward (2022) remarked that “a common theme across these motives over time is the external representation of internal concepts, thoughts, and processes.” That includes help in coping with traumatic experiences (Stirn et al., 2011), and claiming ownership of one's own body (Atkinson, 2004). Whatever the reason, any such visible and durable changes to the body constitute the transmission of information across time, and thus are a form of memory. Body modifications incidentally carry information from the past; they show that a person's body has undergone changes, and is now different from how it once was.

The superordinate phrase “body alteration” was used in a review by Weiler et al. (2024) to encompass both body modification and more temporary “body decoration” such as the application of cosmetics (makeup), a widespread and ancient practice often applied to the face. Weiler et al. identified the main mental functions served by body alteration as aesthetics and group affiliation (with the latter including individuality, resistance, personal narrative, physical endurance, and sexual motivation). Makeup may also be used to obscure visible markings on the skin such as blemishes, bruises, or bags under the eyes from lack of sleep. In this way, it is a kind of manual overwriting of the memory information carried in the skin. Makeup is also readily removed, making it one of the easiest forms of memory to erase.

##### Tattoos

Tattoos in particular can be made specifically for memory purposes, and can last a lifetime. Klug et al. (2024) surveyed 161 German adults with tattoos and found that a majority of tattoos were inspired by unique life events, covering themes of personal growth, relationships, leisure activities, losses, or diseases. They concluded that “tattoos uniquely allow for the physical embodiment of autobiographical memories, indicating that engraving significant life events in the skin aids in reflecting on one's life story.” In short, a tattooed body can tell a story, to others and to the self (Boszorád, 2019; Naude and Naude, 2024). That said, tattoos may sometimes be regrettable reminders of poor choices or past relationships (Houghton et al., 1996; Rivera, 2021). Tattoo removal or coverup is possible, as a form of intentional forgetting.

One clearly memory-oriented purpose of tattoos is to memorialize lost loved ones, for example depicting the name or likeness of the deceased, or a visual theme related to them. Davidson and Duhig (2016) found that such memorial tattoos served several purposes: continuing a bond with the deceased, making the deceased a permanent part of oneself, embodying grief, facilitating conversation with others about the deceased, and visually representing the change that one went through in response to the loss (see also Samuel, 2011).

While the above examples of tattoos comprise a kind of episodic memory inked onto the body, it is also possible for tattoos to act as semantic memory. For example, tattoos can be used to convey important medical information such as diabetic status (Kluger and Aldasouqi, 2013), or to depict useful information such as formulas, conversion tables, rulers, or even QR codes that link to further information. There are temporary tattoos (e.g., henna) that may last for days, and there is the simple act of writing on oneself with a pen. In a currently unpublished study, I surveyed undergraduates who wrote on their hands and found that the main two reasons for doing so were memory and boredom.

Finally, there is a kind of spacing effect in getting tattoos, such that a delay of several weeks is optimal between multiple sessions (encoding), so as to give time for healing and pain management, and not overstress the immune system.

#### Nails

The tips of human fingernails and toenails carry information about recent interactions with the environment. For example, cracks or chips indicate recent damage. A filed edge indicates recent grooming. The duration of such memory is typically on the order of days. The length of the free edge of a nail indicates the amount of time since it was trimmed. The entire nail bed carries information for longer. The maximum duration of this memory is on the order of months, with complete growth of the nail taking about 6 months for fingernails and 10–18 months for toenails (Slotnick and Nriagu, 2006; Yaemsiri et al., 2010). When a nail is stressed due to injury or infection, white spots may appear (leukonychia), and the current position of such spots on the nail indicates how long ago the stress was. Additionally, the nail holds biomarkers of exposure to toxic chemicals (Slotnick and Nriagu, 2006). The condition of the nail is also affected by nutrition and disease (Pasch et al., 2024), as well as occupation (Kanerva, 2000; Ronchese, 1948). Nails may be decorated too, a common form of body modification.

#### Bones and teeth

Human bones and teeth carry a great deal of information about the history of an individual. The fields of human osteoarchaeology and forensic anthropology specialize in deciphering such information, either to understand the lives of people long ago, or to solve criminal cases, respectively. What kind of information can be retrieved from bones as memory? Sex can be determined by characteristics of pelvic bones and skulls. Age for children can be determined by teeth. Age determination for adults is more challenging but can involve macroscopic and microscopic analysis (e.g., histological examination). The composition and mechanical properties of bone vary with aging (Boskey and Coleman, 2010). As one example, osteoporosis, a bone disease associated with aging, causes reduction in bone mass. Height (stature) can be inferred by using the length of, say, a femur, in a regression analysis based on a database (Klepinger, 2006, p. 80–82).

Previous injuries and diseases can be evident in bones (Klepinger, 2006, Chapter 9; Nikita, 2017, Chapter 8), including: abnormalities in development (e.g., scoliosis); diseases such as scurvy, rickets, osteoporosis, or tumors; and injuries such as fractures, blunt trauma, cuts, or gunshot wounds. Patterns of activity in life can be inferred by analyzing entheseal changes (alterations to the surface of bones where tendons attach, influenced by repetitive activities) and cross-sectional geometric properties of bones (affected by mechanical loading during life; Nikita, 2017, Chapter 7).

When it comes to teeth, which are generally the most well-preserved of human remains over time, patterns of decay and wear can reveal diseases and diet even in ancient specimens (Forshaw, 2015). For modern remains, teeth can be used to identify an individual by matching to dental x-ray records. For living humans, x-rays and dental exams reveal the health of a person's teeth and gums, and reflect the person's experiences and oral hygiene behaviors. A chipped tooth is a clear sign of a previous injury, and thus analogous to an episodic memory. Poor oral hygiene can be a warning sign of mental disorders such as depression, anxiety, or dementia (Torales et al., 2017). Bruxism is the habitual grinding of teeth, which yields a pattern of wear and is associated with anxiety (Torales et al., 2017).

Human bones and teeth are the most durable and longest lasting form of memory in our body, and such remains have been discovered dating back to the very dawn of our species (ca. 200,000 years ago), and even earlier to our pre-human ancestors (White et al., 2009). Bones and teeth can even be used to extract DNA (genetic memory; Rohland and Hofreiter, 2007).

#### Muscles and movement

The common phrase “muscle memory” is a bit of a misnomer; it just refers to well-learned motor skills that can be performed without conscious effort (American Psychological Association, n.d.-b). In other words, it is procedural memory, a form of implicit long-term memory, which is stored in the brain. However, it is true that skeletal muscles, which enable voluntary movement, grow when exercised. Thus, muscle mass, strength, and range of motion are records of past experience. The mere presence of large muscles indicates prior exercise.

Muscles also exhibit **savings in relearning**, analogous to memory in the brain. When muscles are exercised, the individual muscle cells (fibers) tear; when these tears are repaired, the fibers grow and also gain extra nuclei. These extra nuclei remain in the fibers even during periods of prolonged inactivity, and will accelerate later muscle growth. That is, if a bodybuilder bulks up their muscles once, then stops exercising for a while, they will be able to bulk up again more quickly for having done it the first time (Blocquiaux et al., 2020).

Kinesiology is the study of body movement. The way someone moves can also reflect past experiences. For example, a person's gait (pattern of walking) can be influenced by disease, injury, aging, or neurodevelopmental disorders, and thus gait analysis is a useful clinical tool (Hulleck et al., 2022). Posture, the position of one's body parts in relation to each other, also carries information. Postural deviations can be influenced by lifestyle (e.g., occupational demands, habit), disease, or injury (Lippert, 2006, Chapter 20). Even a person's idiosyncratic mannerisms and body language likely reflect their history of movement.

#### Voice

The human voice is a sound made by expelling air from the lungs that is vibrated by the vocal cords in the larynx and further modified by the tongue, palate, and lips. The versatility of voice is what enables spoken language. The language one speaks is a function of what one has learned in the past. A native language is learned by infants from exposure to, and interaction with, adult speakers. Additional languages can also be learned later in life. However, the phonemes (language sound units) that one is capable of creating are typically limited to what one learns as a child. One's language and voice are a kind of record of experience, with even regional accents and dialects indicating where one has spent time living (Clopper and Pisoni, 2007).

Pitch carries information too. The larynx grows when exposed to testosterone, deepening the pitch of the human voice. This happens during puberty, especially for males, and during testosterone therapy as part of gender-affirming care (Cler et al., 2020). Thus, deepness of the voice can act as a developmental signifier.

The current characteristics of one's voice also carry shorter-term information. For example, a hoarse voice (dysphonia) can indicate recent overuse, sickness, or smoking, usually within the past days or weeks (Reiter et al., 2015). The smell of one's breath also reflects the recent past, such as ingestion of food and drink, smoking, and dental hygiene or lack thereof.

#### Digestion and excretion

The contents of the stomach, intestines, and bladder are a direct record of what someone has recently ingested. For solid food, this memory lasts ~1–3 days between eating and defecation (whole gut transit time; Lee et al., 2014; Mikolajczyk et al., 2015). For liquids, the memory is much shorter, as little as 5 min between drinking and urination if one is already fully hydrated, up to hours if not. Stool, urine, and saliva carry information not only about what someone has ingested, but also about their overall health and recent exposure to drugs, heavy metals, toxins, or pathogens. Such information is retrieved via laboratory tests (Provan, 2018).

The intestines are host to many commensal bacteria (our microbiota) which help us digest nutrients and also aid the immune system (Martín et al., 2013). The microbiota also interact with the central nervous system, with the microbiota–gut–brain axis playing a role in homeostasis and possibly mental disorders (Cryan et al., 2019). The colonies of gut bacteria are first established (encoded) as early as fetal gestation, and boosted by exposure to the mother's vaginal and fecal bacteria during vaginal birth, and the general environment after birth (Thursby and Juge, 2017). Commensal gut bacteria can be lost (forgotten) due to antibiotics or conditions such as ulcerative colitis, but can be restored (re-learned) via fecal transplant (Rossen et al., 2015).

#### Blood

Blood is a vital fluid in the human body that consists mostly of plasma, red blood cells, white blood cells, and platelets (Dean, 2005). Blood is a vector for immunological memory, transporting memory B and T cells, natural killer cells, as well as antibodies. A complete blood count test (CBC) reveals information about the body's current and recent health. For example, an elevated white blood cell count can indicate current or recent activity of the immune system in response to infection. In terms of genetic memory, blood type (e.g., A, B, AB, or O) can be classified by the types of molecules (antigens) on the red blood cells, and is inherited from parents. Blood acts as a relatively short-term form of memory for drugs of abuse, with detectable traces lasting from about 12 h for cocaine to about 48 h for methamphetamine (Verstraete, 2004), much shorter than the chemical memory stored in hair. Other blood tests, acting as a kind of retrieval mechanism, can detect a variety of other substances in blood, including hormones indicating pregnancy, cortisol, vitamins, minerals, heavy metals and other toxins, glucose (important to monitor for diabetes), electrolytes, molecules indicating cancer, molecules indicating liver or kidney function, and markers of infection due to bacteria, fungi, or viruses (Provan, 2018).

#### Reproductive systems

The presence of secondary sexual characteristics indicates that an individual has experienced sex hormones, either through puberty or hormone treatment for gender-affirming care.

The female reproductive system shows decreasing fertility with age (Pal and Santoro, 2003), ending with menopause and the depletion of egg cells. But it also shows memory in the form of priming, such that the duration of labor tends to be shorter for second and subsequent births (Friedman, 1978; Roediger, 2003). Additionally, the risk of pre-eclampsia (including high blood pressure) decreases in additional pregnancies with the same father (Li and Wi, 2000), possibly driven by the mother's immunological memory to antigens from the first fetus. There is even evidence that a mother's body retains fetal cells of a first pregnancy which can be passed on to the fetus of subsequent pregnancies, resulting in expanded immunological tolerance in those fetuses, and potentially even in further generations (Kinder et al., 2017). Furthermore, the cyclical nature of the the menstrual cycle itself implies an approximately monthly memory system.

Male fertility also decreases with age (Ford et al., 2000). In addition to that, the male reproductive system shows a shorter-term form of memory in the refractory period, the delay before a second erection or ejaculation is possible. Surprisingly little published data exist on the duration of the refractory period (Levin, 2009), but one study (Ekmekçioglu et al., 2005) found a mean of 19 min in a group of 22 men aged 19–47 (*M*_*age*_ = 24). Even less data exist for the common claim that the refractory period increases with age, but one study (Bhat and Shastry, 2019) did report a correlation of *r* = 0.60 between age and post-ejaculation refractory time. The refractory period of the male sexual response is a kind of anti-priming, in that orgasm is impeded by recency rather than facilitated by it.

Female sexual response includes the possibility of multiple sequential orgasms without a refractory period (Darling et al., 1991; Gérard et al., 2021), which is very rare for males (Wibowo and Wassersug, 2016). Although published data are very sparse, two case studies of women reported that an initial orgasm potentiated subsequent orgasms which were more quickly and easily achieved but less intense (Bohlen et al., 1982; Shtarkshall et al., 2008). Thus, the female sexual response can exhibit recency priming.

#### Fat

Excess calories consumed are stored (encoded) in fat cells, which comprise adipose tissue throughout the human body. This reservoir of energy is a kind of memory of past consumption. When energy requirements exceed current consumption, the body can retrieve this memory by releasing triglycerides (fatty acids) into the bloodstream (Rosen and Spiegelman, 2014). Thus, fat forms a clear long-term memory system, with encoding, storage, and retrieval processes. Interestingly, the capacity of this memory system is practically unlimited: the body can always make more and larger fat cells. Scherer (2006) remarked that “adipose tissue is the only organ with unlimited growth potential at any stage of our life.” A rapid and extreme increase or decrease in adipose tissue can be a marker of an eating disorder, such as binge eating or anorexia, respectively. Thus changes in body fat can reflect recent disordered eating behavior. Fat cells also appear to be a storage repository for other chemicals and drugs for long periods, potentially years (Levisky et al., 2000; Cecchini and LoPresti, 2007).

#### Lungs

The functioning of the lungs can be marked for long durations, potentially across decades, by experiences such as respiratory illness, smoking, and exposure to pollution. Function can also be improved by regular aerobic exercise. In the shorter-term, a cough may persist for weeks after respiratory illness. In the even shorter-term, being out of breath is an indicator of very recent exertion. Each of these constitute memory.

The act of breathing itself shows a characteristic called **hysteresis**, which is when the state of a system depends on its history. The relationship between pressure and volume (called compliance) is different for inhalation vs. exhalation (Binks, 2022, pp. 15–18). For example, lungs half-full of air while breathing in have higher pressure than lungs half-full of air while breathing out. Hysteresis is a general property found across a variety of biological and physical systems, and at its root can be considered a kind of memory.

#### Body-based numerical representation

Parts of the body can be strategically used to temporarily represent information. The most common example is counting with one's fingers. This is a kind of offloaded working memory, and it appears to be a universal cognitive strategy. Göbel et al. (2011) argued that Paleolithic cave paintings featuring sequences of hand outlines show that finger counting dates back at least 27,000 years. Finger counting is an example of embodied cognition (Wilson, 2002), and it has been studied with respect to the topic of “spatial numerical associations” (Fischer and Brugger, 2011). Curiously, Dupont-Boime and Thevenot (2018) found that children with low working memory capacity were less likely to use their fingers in an addition task. They suggested this is because even the very discovery of finger counting is demanding on working memory in the brain, especially the more helpful and elaborate “min strategy” (starting with the larger number and then adding the smaller); thus finger counting strategies should be explicitly taught to children.

## Expansion 3: individual vs. collective memory

**Figure 6:**
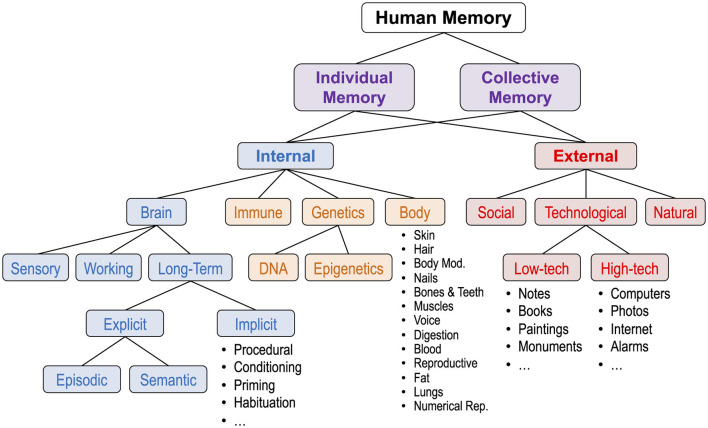
Third expanded taxonomy of human memory. Changes from the second expansion are as follows. There is now a superordinate distinction between individual and collective memory. Natural external memory is added. Technological external memory is now defined as information stored in the human-made environment (no longer the inanimate environment).

The taxonomies presented so far are all oriented around a single human. The memory systems of an individual's brain (Figure 1), the information available to an individual outside of themselves (Figure 3), and the information stored elsewhere in that individual's body (Figure 5). Although an individual mind is the traditional unit of analysis in cognitive psychology, it is also true that human beings are inherently social animals, and exist in the inextricable context of other humans. Our interactions and lives with each other manifest as a culture. Thus, the last expansion I propose to a taxonomy of human memory adds a superordinate level of analysis distinguishing between individual memory and collective memory, as well as adding a new natural category of external memory, as shown in Figure 6.

### Collective memory

Collective memory concerns the shared memories of a group's history that are important to the group's identity.^8^ Collective memory is an umbrella term with varying definitions, especially across disciplines (Abel et al., 2017; Hirst and Manier, 2008; Wertsch and Roediger, 2008). Synonyms for collective memory include public memory, cultural memory, social memory, and popular memory. The term cultural heritage is closely related. Collective memory is distributed across: the minds of the individuals in a social group; their oral and written narratives and folklore; architecture and monuments; rituals, festivals, holidays, and traditions; and songs and other public symbols. The inanimate physical parts of collective memory (e.g., art, text, tools, furniture, clothing, buildings) can be called *material culture*, a term from archaeology which is roughly synonymous with what I call technological external memory (Finley et al., 2018, p. 122; see also Heersmink, 2023 for a discussion of “cultural artifacts”). Collective memory may serve several functions, such as: creating and maintaining group identity, providing narrative structures (schemas) to interpret past and current events, and guiding the group's future (e.g., avoiding past mistakes; Heux et al., 2023; Hirst et al., 2018). As Lehrner and Yehuda (2018) said:

Many cultural practices and rituals function to transmit historical knowledge and experience across generations. … Transmission of cultural memory through rituals, symbols, and practices serves to transmit learning and meaning, to allow future generations to understand the world and to respond adaptively. (pp. 1763, 1772)

Different groups may remember the same events differently. For example, Abel et al. (2019) found both similarities and differences in how people from 11 different countries remembered the important events of World War II. Russians focused more on events in their sphere (e.g., Battle of Stalingrad) and Americans focused more on events in theirs (e.g., bombing of Pearl Harbor). On a smaller scale, fans of two rival football teams who watched the same video of a game came to different conclusions about which side played rough (Hastorf and Cantril, 1954). The malleability of individual memory in the brain is what enables this (Coman et al., 2009).

Different groups may also show different cultural approaches to collective remembering. Schwartz et al. (2005) found that American college students felt less responsibility for their country's past wrongdoings than did Japanese college students. This may reflect the differences between an individualistic culture that encourages cutting ties with the past vs. a collectivist culture that feels more connection to its ancestors and history. Wang (2008) also explored how cultural context shapes collective memory, for example showing that Chinese adults were far more likely than European adults to make spontaneous references to historical events when recalling autobiographical memories. Wang (2021) presented a culturally saturated mnemonic framework to capture the ways culture shapes individual human memory.

The capacity of collective memory is even more unlimited than the capacity of the long-term memory of the individuals in the group, expanded further by technological external memory. The duration of collective memory is likewise indefinite. Assmann and Czaplicka (1995) suggested that memories transmitted within a group via everyday communication may last 80–100 years (3–4 generations), while memory that becomes fixed outside of individual minds, in textual archives, monuments, and societal practices such as rites and commemorations, may last an unlimited time. In a sense, collective memory may long outlive every last member of a group, in the form of material culture, such as artifacts, ruins, or even ancient cave paintings. This is how we know about lost societies of the past (Hurcombe, 2014). At the same time, orally transmitted knowledge can also endure for potentially thousands of years, as I will review when considering Collective Memory and Social External Memory.

Among the various ways of conceiving collective memory, Dudai (2002) argued for three components: a shared body of knowledge, an attribute (a schematic narrative of a group of people), and a process (e.g., disputing and reshaping interpretations of the past). Note that the body of knowledge can extend to events beyond the individuals' personal experience, such as the founding moments of a country (cf. postmemory, Hirsch, 1997, p. 22), alongside events in living memory. Abel et al. (2017) reviewed theory and data for each of those three components, including **collaborative remembering**, which can be thought of as a process of collective memory when people in a small group work to remember something together, often by discussion (Harris et al., 2008; Rajaram and Maswood, 2018; Weldon, 2000). Hirst and Manier (2008) noted that collective memory can be studied from an epidemiological approach too, as “the transmission of a memory across a network of community members and social resources” (cf. memes, memetics; Blackmore, 1999; Dawkins, 1976; Distin, 2005; Lake, 1998; Shifman, 2013).

To what extent can we differentiate collective memory from *history*? Wertsch and Roediger (2008) described history as an academic endeavor with the goal of creating objective accounts of the past, whereas collective memory is more subjective, tied to the identity of a group, and more often relies on simplified and schematic narratives. Rusu (2013) also contrasted the two, characterizing the ideal of history being etic, analytic, objective, unconcerned with identity, accepting of ambiguity, constrained by archival material, and open to revision, while characterizing collective memory as being emic, synthetic, subjective, concerned with identity, and resistant to change in the face of findings that contradict the narrative.

Research on collective memory took place much more in sociology, history, philosophy, anthropology, and political science before psychology finally joined in (see Barnier and Sutton, 2008 introduction to the special issue of *Memory*). Danziger (2008, pp. 264–274) attributed psychology's tardiness to its intellectual heritage of individualism. The concept of collective memory began in sociology, with the work of Halbwachs (1925/1992), who held that an individual's memories are necessarily reconstructed in a social context, and that individual and collective memory are in constant interplay:

One may say that the individual remembers by placing himself in the perspective of the group, but one may also affirm that the memory of the group realizes and manifests itself in individual memories. (p. 40)

A more recent sociologist, Olick (1999), articulated two competing concepts of collective memory: individualist vs. collectivist. He wrote, “two radically different concepts of culture are involved here, one that sees culture as a subjective category of meanings contained in people's minds vs. one that sees culture as patterns of publicly available symbols objectified in society.” Hirst and Manier (2008) argued for reconciling these two approaches, which they saw as ends of a continuum. I see the two approaches as embodying internal memory vs. external memory, which is why collective memory connects to both of those systems in my taxonomy. Rather than proposing various subsystems for collective memory, I have chosen to simply connect it to the previous taxonomy alongside individual memory. In this way, it lends itself to a revisiting or reinterpretation of the various lower systems.

A note, however, before proceeding. It is important to emphasize that collective memory, like individual memory, must exist in some physical substrate—for example, in the brains of individuals, and/or in material culture such as texts, art, monuments, and libraries (i.e., technological external memory). Even if we say it exists as rituals and traditions, or processes such as collective remembering, those only happen thanks to the brain-driven actions of individuals, as guided by material culture. The concept of collective memory should not be mistaken for metaphysical notions of inherited racial memory, world souls, or Jung's idea of a collective unconscious, which at best was simply a shared tendency of humans to think in certain ways, and at worst was an unfalsifiable supernatural dimension (e.g., “The collective unconscious comprises in itself the psychic life of our ancestors right back to the earliest beginnings. It is the matrix of all conscious psychic occurrences…” Jung, 1929/1960, ¶230).

### Collective memory and neurological memory

Whereas individual memory is about the ways that information is encoded, stored, and retrieved within an individual's lifetime, and with respect to that individual, collective memory is about the ways that information is transmitted across individuals and across lifetimes. The collective shapes the individual, and the individual constitutes the collective, in part. I will now revisit the various subsystems of neurological memory (i.e., memory in the brain) with respect to the lens of collective memory.

#### Collective memory and sensory memory

Echoic memory, which is sensory memory for auditory stimuli, can retain sound information for up to several seconds. This is essential for language comprehension, allowing the individual to string phonemes into words and words into phrases. This enables conversation, which is essential to collective memory. There is some evidence that poorer echoic memory impairs learners' listening comprehension in a foreign language (Greenberg and Roscoe, 1988).

#### Collective memory and working memory

The limited capacity of working memory is the major bottleneck of human cognition. When several people are collaborating on a learning or problem-solving task, they may be able to form a collective working memory by sharing knowledge and distributing elements of the task across group members (Kirschner et al., 2018). In a very simple everyday manner, you may ask someone else to temporarily remember something for you (e.g., an address) while you are engaged in some other task (e.g., driving), thus pooling your working memory resources.

#### Collective memory and long-term memory

When researchers write about collective memory in individuals, they are typically talking about long-term memory in the brain. As per Figure 1, there are several subdivisions to long-term memory, and each of these gives a different view on collective memory. Manier and Hirst (2008) similarly applied the subdivisions of long-term memory to a taxonomy of collective memory.

##### Collective memory and long-term memory: explicit: episodic

When a group of people have a shared experience, their individual episodic memories of that experience—considered in aggregate—are collective episodic memory (Manier and Hirst, 2008). This may be on a small group scale, such as a family's shared memories of a vacation. Reminiscing about shared family memories can shape and connect the individual episodic memories as well as maintain group identity (Bietti, 2010; Fivush, 2008). In some cases, twin siblings may even dispute ownership of the experiences they remember (Ikier et al., 2003).

Collective episodic memory may be on a large societal scale too, such as flashbulb memories of learning about a national disaster like the 9/11 terrorist attacks in New York City, 2001. Research on flashbulb memories of that event has shown that peoples' confidence in their memories exceeded the accuracy and consistency of the memories after 8 months (Talarico and Rubin, 2003). After 10 years, Hirst et al. (2015) found that people showed marked forgetting of reception details (i.e., how they found out about the attacks) in the first 3 years, followed by stabilization, but still with elevated confidence. They also found evidence that media attention and ensuing conversation served to correct memory errors about details of the attacks.

Different groups may hold different collective memories not just of history but also of experiences they lived through. For example, the 1992–1995 conflict in Bosnia and Herzegovina is remembered differently by Bosnian Muslim people vs. Serbian people vs. Croatian people (Džidić and Dzidic, 2014; Martinovic et al., 2017; Palmberger, 2016).

Collective episodic memory changes over time, with each generation of people having their own sets of salient shared memories. For example, the Pew Research Center (1999) polled Americans of different ages and found that the explosion of the Challenger space shuttle (1986) was a top five remembered event for those aged 18–34, but not for those aged 65+. Beyond just the progression of generations, Schwartz et al. (2005) remarked that “we remember the past as members of society, [and] as the structures and values of society change, different parts of the past gain and lose relevance.”

Nostalgia, a wistful longing for the past, can be a potent feature of collective episodic memory. The past to which people may wish to return varies across time and groups. For example, many develop passion for the media technologies and content (external memory) of their formative years (Bolin, 2016). National nostalgia for “the good old days” as framed in political discourse can foster angst about a country's present and future (Ionescu et al., 2023).

Individual autobiographical memory (which has both episodic and semantic components) develops in a social and cultural context (Nelson and Fivush, 2004; Reese and Fivush, 2008) and one's autobiographical narrative can be shaped by the way in which their family reminisces (Fivush, 2008), which varies across cultures (Wang et al., 2000). Thus, the collective shapes the individual, just as the individual constitutes the collective.

##### Collective memory and long-term memory: explicit: semantic

Semantic memory is for facts and general knowledge, rather than episodes tied to a specific time and place. In collective memory, this is the shared body of knowledge common to members of a group, passed down through education, media, and cultural practices. This could include knowledge of: history (e.g., Texans' knowledge of the Alamo, or French peoples' knowledge of the Storming of the Bastille), symbols such as flags and anthems (e.g., the rainbow pride flag for the LGBTQ+ community, the song *Lift Every Voice and Sing* for the African American community), the rules and standards of a group (e.g., speed limits, units of measurement), professional knowledge (e.g., anatomy and physiology memorized by medical doctors), religious knowledge (e.g., the text of the Quran memorized by Muslims), culture and media (e.g., The Beatles for the Baby Boom generation, Michael Jackson for Gen X), sports and leisure (e.g., the names and positions of players known to fans of a soccer team), the natural world (e.g., bird songs recognized by birdwatchers, medicinal plants used by Indigenous Peoples), and so on.

Manier and Hirst (2008) distinguished between what they called “lived semantic memories” which is knowledge for unexperienced events in one's lifetime and “collective distant semantic memory” which is knowledge for events before one's lifetime. They argued that knowledge of events in living memory has greater resonance (see also “demographic memory,” Gaddy, 2023). Here, I would like to suggest an alternative idea that I think is more useful, the idea of “event memory” (Rubin and Umanath, 2015) as “the mental construction of a scene, real or imagined, for the past or the future.” This encompasses historical memories of events that an individual did not personally experience, but that nevertheless have episodic-like properties, such as Neil Armstrong walking on the Moon, or Abraham Lincoln giving the Gettysburg Address, and so on (see also “event model”, Zacks, 2017). Many foundational myths of a group's collective memory may be characterized as event memories. The concept even includes episodic-like memories of culturally important fictional media, such as a vivid recollection of Luke Skywalker fighting Darth Vader. So we have here an example of the inadequacy of the episodic/semantic dichotomy in explicit long-term memory; however that shortcoming is not exclusive to collective memory, merely highlighted by it.

Semantic memory also includes several generalized memory structures that are useful for organizing information in the brain, such as schemas. A schema is a kind of mental template or framework of expectations, built from experience, that serves to direct attention, and assist with encoding and retrieval/reconstruction of episodic information (Brewer and Nakamura, 1984). At the level of collective memory, several scholars have written of **schematic narratives** which are generalized forms of narratives that mediate collective memory by shaping specific narratives. For example, Wertsch (2002) proposed a “triumph over alien forces” narrative template in which Russia is the hero that expels invaders. This schematic narrative shapes specific narratives in Russian collective memory of several different conflicts, from the “Mongol Invasion” in the 1200s, to the “Patriotic War” against Napoleon in the 1800s, to the “Great Patriotic War” against Hitler in the 1900s. In general, **stories** are a powerful and useful memory structure for humans (Schank and Abelson, 1995), likely because stories are inherently social and we are a social species (Read and Miller, 1995).

Another general memory structure is the **cultural life script**, a culturally shared representation of the timing of major transitional life events (Berntsen and Rubin, 2004). These scripts vary across cultures, and shape how individuals in a culture recall their own life experiences and choose to tell their life stories (Berntsen and Bohn, 2009).

**Social norms** (Sherif, 1936) are another kind of general memory that can be considered part of the collective memory of a group, although they perhaps straddle the line between explicit and implicit long-term memory. Social norms include expectations for behaviors in particular contexts and based on classifications such as gender (i.e., gender roles). Such norms vary across groups.

##### Collective memory and long-term memory: implicit

Manier and Hirst (2008) classified community traditions, practices, and rituals as **collective procedural memories**. Procedural memory is one instance of implicit long-term memory, which refers to skilled performance that is often not able to be verbalized. Consider for example the forms of greeting across cultures, such as shaking hands vs. bowing, or the norms for personal space and standing in lines (queues). Manier and Hirst (2008) wrote:

Rituals and traditions, or more generally, procedural memories, can serve as mnemonic tools that shape the collective identity of their practitioners, collectively reminding them of declarative memories. The celebration of Mass is intended to remind parishioners of Jesus's crucifixion. (p. 259)

Lehrner and Yehuda (2018) provided another religious example:

Many cultural practices and rituals function to transmit historical knowledge and experience across generations. The Jewish Passover seder gathers family and friends for a meal during which the biblical story of the escape from bondage in Egypt is retold and freedom is celebrated. Children are specifically instructed to consider what the story means to them and about them. (p. 1763)

The observance of Ramadan by Muslims involves a month of fasting and reflection toward personal growth and connection with God. It includes recitation of prayers, one form of which is called dhikr (remembrance). Prayer beads may be used for counting of repeated prayers, a form of external memory similar in function to rosary beads used in Catholic prayer. The five daily prayers, salah, involve a sequence of movements called a rak'ah, which is repeated a different number of times at different points in the day by Muslims all over the world, a clear form of collective procedural memory, and one that has been shaped and maintained by oral tradition because the Quran does not give specific instructions. A secular example of ritualized behavior in the United States would be standing up, removing headwear, and singing the national anthem at the start of a sports game.

Because collective procedural memories are implicit and specific to a culture, they can be a source of culture shock or cultural misunderstandings. Simple automatic behaviors that are considered polite in one culture may be considered rude in another, for example the amount of eye contact used in conversation, the number of hands used to give or receive objects, or pointing with one finger. And because these are implicit memories, hosts may not even think to explain them to visitors. To the extent that customs and traditions are in fact verbalizable, if one takes the time to think about them, they are perhaps a blend of semantic and procedural memory.

#### Collective memory and long-term memory: processes

Here I will consider parallels and differences between the *processes* of collective memory and individual long-term memory.

##### Retrieval and spacing

We know that when an individual retrieves a long-term memory, whether episodic or semantic, that memory is strengthened (the retrieval practice effect, or testing effect; Roediger and Butler, 2011; Rowland, 2014). How does this play out at the level of collective memory? Roediger et al. (2009) contended that repeated retrieval spaced over time serves to maintain collective memories. This may happen in the form of annual holidays, reunions, rituals, or sites of historical remembrance (Winter, 2009, 2010). The choices a society makes do not just maintain collective memory, but shape it too. For example, in the United States, it took years of campaigning before the federal government in 1983 established Martin Luther King Jr. Day as a national holiday, annually elevating awareness in collective memory of the Civil Rights movement for racial equality in the 1950s−1960s (National Museum of African American History and Culture, n.d.). The 2021 establishment of Juneteenth as a national holiday commemorating the end of slavery served to elevate an African American celebration of freedom to national consciousness, but not without political machinations (Scott and Jordan, 2022). Indigenous Peoples Day, started in 1992, continues to gain awareness and increased observance as a counter-celebration to the federal holiday of Columbus Day, which detractors see as celebrating the genocide of Native American people by Europeans (Fadel, 2019). These examples illustrate that collective memory is often in contention, and that annual observances can shape that memory through the power of reminding (retrieval practice). Furthermore, the fact that such events are spaced 1 year apart exploits the spacing effect (Cepeda et al., 2008; Dempster, 1988), by which memory is particularly strengthened from spaced rehearsal.

##### Repression, recovery, and genocide

There are some very interesting differences in the workings of collective memory vs. individual memory in the brain. For example, **repression**. Repression is an idea in psychoanalysis developed and popularized by Freud: an automatic defense mechanism that can banish painful experiences, thoughts, feelings, or desires to some place beyond conscious awareness.^9^ “The essence of repression lies simply in turning something away, and keeping it at a distance, from the conscious” (Freud, 1915, p. 147). In memory research, this concept has mainly been considered in the context of repression of memories of traumatic experiences. In the 1990s, during a feud called the “memory wars” (Lynn et al., 2023) that spilled from academia into public media, legislation, and judicial matters, some clinical psychologists championed the idea that repressed memories of childhood sexual abuse were real and could be recovered through therapy, resulting in widespread reports of recovered memories even including satanic ritual abuse. Cognitive psychologists (e.g., Loftus, 2005) countered that empirical evidence showed how the very therapies used to supposedly recover repressed memories (e.g., suggestive questions, hypnosis, guided imagery, dream interpretation) may have been instead creating false memories (Ceci and Loftus, 1994). There was also the lack of corroborating evidence for abuse recovered through suggestive therapy (Geraerts et al., 2007). Furthermore, other more mundane explanations are also plausible for the apparent recovery of supposedly repressed memories, such as regular forgetting, reluctance to talk about abuse, not recognizing abuse for what it was until an adult perspective was gained, and the forgot-it-all-along effect in which someone forgets that they used to remember something (Geraerts et al., 2009; Otgaar et al., 2022). Finally, evidence shows that people who experience well-documented trauma, even as children (Malmquist, 1986), generally *do* remember the events rather than repressing them. Indeed, a hallmark of post-traumatic stress disorder (PTSD) is intrusive memory of the trauma (Brewin, 2014). Thus, the idea of repressed memories has been scientifically discredited (Loftus and Ketcham, 1996), despite continued belief in it by the public and some clinicians (McNally, 2023; Patihis et al., 2014).

However, all of the above applies to the ideas of repression and recovery for individual human memory in the brain. Could a mechanism like repression exist at the level of collective memory? Yes. A fascinating case study is found in the Spanish Civil War (Baldini, 2018). This war took place from 1936 to 1939, ending with the Nationalist faction winning and General Francisco Franco taking power and ruling as a dictator until 1975. Franco's regime repressed, or “cleansed” the collective memory of the losing Republican side in several ways: exiles, executions, imprisonment, destruction of books, censorship of the press, and taking absolute control of education so that only the Nationalist view of history was taught (Mauri-Medrano, 2019). Terror gripped the populace, as no one was allowed to speak their knowledge of how events had really occurred. This led to “the wall of silence that the Franco regime raised to hide facts and repressive activities that almost everybody knew, but no one dared talk about it.” (Grana-Gil et al., 2014). After Franco died in 1975, Spain transitioned to a democracy, but a 1977 Amnesty Law both returned those who had been exiled and guaranteed impunity for those of Franco's regime who had committed crimes. This was part of the Pact of Forgetting agreement by the political parties to essentially sweep the whole Civil War and dictatorship under the rug to be forgotten (Encarnación, 2014). But by the early 2000s, young people sought stories from their grandparents' generation of the “forgotten” period of history (a cultural amnesia), so they could reclaim a cultural narrative in which to better establish their own identities. An example of the resulting cultural output is a book, *The Sleeping Voice* (*La Voz Dormida*) by Chacón (2006). Finally, in 2007 the government passed the Historical Memory Law to, ideally, lessen the repression of memory.

The example of the Spanish Civil War shows that processes of repression and recovery are possible in collective memory. Contestation and dispute are fundamental to collective memory, and governments can play a major role in shaping collective memory in ways that even override personal experience, such as presenting official narratives and silencing other narratives. Wertsch (2002, Chapter 4) gave another example of state production of official narratives (the Soviet Union), and Jelin (2003) covered state repression in several South American countries. DeGloma (2015) called disputes over collective memory “mnemonic battles” and proposed they take three forms: disputes over the existence, nature, and relevance of the past. In a sense, those in power can inflict false memories onto the populace, which may become official history. Kansteiner (2002, p. 186) observed that “nations can repress with psychological impunity.”

In the case of Spain, some of the repressed collective memories were able to be recovered; but this is not always the case. The original definition of “genocide” by Raphael Lemkin in 1943 was “the destruction of a nation or of an ethnic group” and it included not just killing but also “disintegration of the political and social institutions, of culture, language, national feelings, religion, and the economic existence of national groups…” (p. 79). That is, genocide included destruction of a group's collective memory. During the Genocide Convention in 1948, the UN omitted the cultural components of Lemkin's definition, in part due to concerns by powerful countries like the United States about their own culpability (Bachman, 2022, pp. 63–66). Because cultural genocide *has* been carried out by the United States, decimating the collective memories of groups such as Indigenous Peoples by means of forced assimilation, relocation, control of education (e.g., American Indian boarding schools), and suppression of language and beliefs (Kingston, 2015; Mako, 2012). Additionally, enslaved African people brought to America also suffered cultural genocide through the loss of their languages and cultures. Although many Indigenous Peoples do still survive, as do African Americans, some lost memories cannot be recovered. In a perverse additional twist, denial that genocide even happened is often a struggle in collective memory (Huttenbach, 2000).

##### Consolidation

In a fascinating book, Anastasio et al. (2012) argued that similar principles underlie the consolidation of new memories in both individual neurological memory and collective memory. They proposed a model of consolidation that applies to both levels, consisting of three components: a buffer (for temporary storage of unconsolidated information), a relator (for linking new information with existing memory networks), and a generalizer (to abstract and simplify memories, for example using schematic narratives).

##### Forgetting

Just as forgetting is not necessarily always bad in individual memory (Bjork, 2014; Karpicke and Coverdale, 2020), it can also serve multiple purposes in collective memory. Connerton (2008) wrote of seven different kinds of forgetting in collective memory: repressive erasure (which I have just discussed), prescriptive forgetting, forgetting that is constitutive in the formation of a new identity, structural amnesia, forgetting as annulment, forgetting as planned obsolescence (which could apply to advances in technological external memory), and forgetting as humiliated silence. The particular agents involved range from individuals to small groups to nations. I might add the concept of institutional memory loss, which can happen when members of an organization leave and take with them their semantic and procedural knowledge relevant to the operation of the organization (Bowker, 1997). Linde (2008) explored how narratives shape collective memory and future trajectories of organizations. Linden and Rutkowski (2013) argued for beneficial forgetting at both the individual and collective levels.

Collective retrograde amnesia was considered by Anastasio et al. (2012, Chapter 9) with the example of the Chinese Cultural Revolution (1966–1976) as a traumatic event for mainland Chinese people. They described a graded memory loss for the suppressed literary and religious works of recent authors, but no loss for long-established cultural knowledge such as Confucianism, Daoism, and Buddhism. Furthermore, they described no collective memory loss for Chinese people living elsewhere during the revolution.

Science is an organized human endeavor toward understanding the universe, and it relies on the accumulation of knowledge, a kind of collective memory (Bowker, 2005). However, even with the advanced databases and access to the scientific literature afforded by the information age, forgetting happens. Zhai et al. (2022) analyzed the pattern of references in the field of Library and Information Science and found that many previously famous works were no longer cited. The reasons for this included obliteration by incorporation (i.e., the information in the original works became standard knowledge in the field), falling out of fashion (i.e., topics or approaches to research that stalled or were abandoned), short timeliness (i.e., the data were only relevant for a short period), and replaced by new knowledge (i.e., concepts that were later superseded). That important works of scientific literature exist but are no longer cited is analogous to the concept of storage strength vs. retrieval strength as described in the New Theory of Disuse for individual neurological memory (Bjork and Bjork, 1992).

##### Availability

Collective memory can show biases just like individual memory can. For example, ingroup inflation is when a group overestimates its own historical significance. Yamashiro and Roediger (2021) argued that this bias is driven by the **availability heuristic** at work in the individuals' minds (Tversky and Kahneman, 1973), whereby they judge a group's contribution to history by relying on how easily they can retrieve examples from their long-term memory. Using a task that asked participants to estimate the percentage historical contribution of three different US states (one of which was the participant's home state), they found evidence in support of this hypothesis. The more knowledge participants could recall about their home state relative to the others, the greater they overstated their home state's influence. Thus, cognitive processes at the individual level can shape outcomes at the collective level.

### Collective memory and external memory

Collective memory spans both internal and external memory. Here I consider how it intersects with the different forms of external memory: social, technological (both low-tech and high-tech), and a newly added form, natural external memory.

#### Collective memory and social external memory

The idea of social external memory, information available to an individual from other people, is inherently collective. Collaborative remembering is when people in a small group work together to remember something, often by discussion (Weldon, 2000). This serves both social and cognitive purposes. Socially, collaborative remembering can serve a variety of functions such as relationship maintenance and creating a shared reality (Pasupathi and Wainryb, 2018). Rimé (2009) identified motives people have for sharing an emotional memory, which included obtaining comfort, strengthening social ties, arousing empathy, and gaining attention. According to Fivush (2008), reminiscing about shared memories is part of everyday interactions within nearly all families (see also Pratt and Fiese, 2004). When a family experiences events together, that can create a collective family memory and facilitate bonding (Jepson et al., 2019; Melvin et al., 2020). Such shared memories support a “feeling of identity among group members” (Bietti, 2010). Remembering life events with a partner can foster intimacy (Alea and Bluck, 2007). At the same time, just as family traditions can be passed down through generations, so can trauma (although evidence here is mixed, Thornberry et al., 2012). At the level of larger groups, traumas transmitted across generations can become a part of the group identity (Volkan, 2001).

What about the cognitive purposes of collaborative remembering? Wegner et al. (1985) proposed the idea of transactive memory in a dyad as “a combination of individual minds and the communication among them” (see also Huebner, 2016). Importantly, for a transactive memory system to work well, group members must have knowledge (a mental model) of the other group members' knowledge; that is, they must have an idea of who knows what. Examples of transactive processes that a dyad may engage in are dividing up memory tasks based on the partners' relative expertise (a differentiated structure; Wegner et al., 1991) or using interactive cueing to jointly remember a shared experience with richer and more emotional detail (an integrated structure; Harris et al., 2014).

There is evidence from laboratory research that collaborative remembering can be both beneficial and detrimental to retrieval of information (Harris et al., 2013; Rajaram, 2011, 2024a). Collaborative inhibition is when a small group working together recalls less than the sum of the same number of people working alone (Marion and Thorley, 2016), possibly due to disruption of individual retrieval strategies (Basden et al., 1997). But having been exposed to things that others remember also gives an additional study opportunity that can help later individual recall (Blumen and Rajaram, 2008). Furthermore, collaborative remembering can provide feedback that shapes collective memories, for example when individuals provide corrections or additions to their shared episodic memories (Roediger et al., 2009). But this can introduce errors too, as shown by research on social contagion of memory, in which participants incorporated false recall answers from a confederate into their own memories tested later (Maswood et al., 2022; Roediger et al., 2001). Coming to an agreement about the past among several people could be seen as analogous to the reconstructive nature of individual memory retrieval, and with similarly mixed results regarding accuracy and completeness.

At a broader scale and longer term than small groups recalling laboratory stimuli, we have **oral tradition**. Oral tradition is the transmission of collective memory across generations through speech and song, with minimal or no use of technological external memory aids such as writing. It includes the stories that groups tell about themselves and the world, as well as a variety of knowledge (e.g., history, law, Traditional Ecological Knowledge). For most of human history, up until the invention of written language some 5,000 years ago, this was the only way to transmit such complex information beyond living memory (i.e., beyond the lifetimes of those who can remember an event firsthand). Oral tradition depends entirely on neurological memory for storage, and oral communication between humans for perpetuation. It takes many forms (genres) such as: legends, myths, folktales, ballads, chants, poems, nursery rhymes, proverbs, and jokes.

Rubin (1995, 2009) studied oral tradition from the perspective of a cognitive psychologist, focusing on three forms: ballads, epic poems, and counting-out rhymes (e.g., “eenie, meenie, miney, mo”). Instances of these forms of collective memory have remained stable over centuries, although not with 100% accuracy by the standards available with technological external memory. In fact, Rubin notes, “the whole concept of verbatim recall requires a record other than human memory; otherwise, it can only mean accurate within the limits of human memory.” (p. 6). The success of oral tradition is particularly impressive given research showing that even recall of simple lists can quickly become distorted across social transmissions (Bartlett, 1932, Chapter VII; Roediger et al., 2014). Rubin (1995) argued that the stable longevity of oral tradition is facilitated by organization of meaning (e.g., scripts, story grammars, and causal chains), spatial and visual imagery, and patterns of sound (e.g., rhyme, alliteration, assonance, rhythm, and music). At the same time, the structures allow for enough variation for performers to adapt to an audience. The process of transmission is facilitated by repeated and spaced practice by the performer, and by performance in special social situations.

Oral tradition is universal, found in cultures across the world, and still to this day offering us threads of continuity with a collective past. Scheub (1985) reviewed oral traditions in Africa, the birthplace of humanity, and how they contributed to written literature. “The African oral tradition distills the essences of human experiences, shaping them into rememberable, readily retrievable images of broad applicability with an extraordinary potential for eliciting emotional responses.” He quoted one San storyteller who said: “A story is like the wind: it comes from a distant place, and we feel it.” Šcigulinská (2015) reviewed parallels in oral traditions of Native American and Australian Aboriginal people, and how those too influenced written literature. However, Hulan and Eigenbrod (2008) argued that conversion to text can obscure the layered complexity of Aboriginal oral narratives. Nevertheless, oral tradition gives us the oldest remaining unbroken links to the past of our species. For example, several Australian Aboriginal groups have passed down stories about the shape of coastlines before they were altered by an ancient rise in sea level. Nunn and Reid (2016) empirically corroborated these stories using modern geographical data, showing that the socially transmitted collective memories had remained accurate for over 7,000 years. Nunn (2018a) related other such ancient memories, including a tale from the Klamath Native American tribe of a volcanic eruption 7,700 years ago. Specialized story-keepers and relative isolation appear to be keys to the extraordinary longevity of such oral tradition.

#### Collective memory and technological external memory

In addition to being stored in the brains of individuals, collective memory is also stored in artifacts of material culture (Finley et al., 2018, pp. 119–123), that is, in technological external memory. Here I will separately consider the intersection of collective memory with both low-tech and high-tech external memory.

##### Collective memory and low-tech external memory

Low-tech external memory, by my definition, is that which does not require a power source to operate. The most ancient evidence of such external memories are marked bone fragments dating back as far as 70,000 years ago (d'Errico et al., 2001). Such objects were likely used to keep a tally of something (cf. tally sticks, Lagercrantz, 1973), but their full meaning has been lost to time. We do not know if they were used individually or collectively. But we do know of later forms of external memory that likely served collective purposes, such as the Ancient Peruvian quipus (or khipus). These were knots tied on a series of small cords, with different types of knots representing numerical values and different colors representing different objects (e.g., types of livestock, soldiers). These aided history and bureaucracy (Tylor, 1870, pp. 156–160; Locke, 1912).

Cave paintings depicting animals date back at least 35,000 years (Aubert et al., 2014). Cave paintings and other forms of rock art likely served collective purposes (Zubieta, 2022; Whitley, 2022), such as rituals (Fonseca-Ibarra, 2018) or repositories of shared meanings (Cupchik, 2016, p. 266). Visual art of all kinds (e.g., paintings, fashion) has remained a staple of human culture and collective memory to this day. Music plays a role too, for example as part of oral tradition, and later as part of mass media.

The invention of written language ~5,000 years ago accelerated collective memory beyond oral tradition, enabling technological external memory to carry complex meaning (Rubin, 1995, pp. 320–325). At first it was used for logistical purposes such as inventory and taxes. But in time history, literature, and religious texts became possible. The oldest written story we still have is the Epic of Gilgamesh (c. 2100 BCE). With the invention of written language, books and scrolls could be made and distributed, albeit slowly. People were able to communicate at a distance via letters, with the added requirement of learning to read and write. All of this gave collective memory an alternative route into the future beside oral transmission, increasing its chances at longevity.

The invention of the printing press around 1440 CE dramatically broadened the reach of the written word to influence and reify collective memory in the centuries that followed. The printed word catalyzed the scientific revolution, starting the collective memory that is the world's body of scientific knowledge (Bowker, 2005). Newspapers were born, and journalism. Libraries and archives proliferated as bastions of public knowledge (Blouin, 1999). Literacy increased.

There are other vectors of collective memory beside written language. For example, there are monuments, memorials, and cemeteries to commemorate people or events. Historical sites (also called heritage sites or sites of memory) can be places to remember ancient civilizations, including world-famous sites such as the Great Pyramid of Giza, Machu Picchu, the Acropolis of Athens, Stonehenge, and the Great Wall of China. Sites of memory can also be places to remember more contested heritage (Maerker et al., 2018), such as the Villa Grimaldi in Santiago, Chile, which was a detention and torture center during the dictatorship of 1973–1990 (Morales et al., 2022; Piper-Shafir et al., 2018). The very architecture of a place and culture serves as collective memory, but is vulnerable to destruction by oppressive forces in acts of cultural genocide. Examples include the destruction of mosques in Bosnia along with the expulsion of the Muslim population by Serbian militant forces in 1992–1995, and the destruction and plundering of ancient cultural heritage sites in Iraq and Syria by the Islamic State in 2014–2015 (Bevan, 2016). Such material culture is sometimes the only traces we have of ancient collectives.

There are also museums housing collections of artifacts as collective memory. Marontate (2005) argued that museums have evolved from special-interest repositories to agents of public culture. Museums can also become contested sites of remembrance, as with the controversy over the display of the Enola Gay on the 50th anniversary of the plane dropping the atomic bomb on Hiroshima (Zolberg, 1996). In modern times, both libraries and museums have become cultural institutions that combine low-tech and high-tech external memory (Bautista, 2013; Borgman, 2000; Robinson, 2012).

In everyday life, humans use both low-tech and high-tech external memory for collective purposes. Paper flyers promote events on community bulletin boards. Yard signs, car bumper stickers, and flags convey group membership and support or opposition for political causes. Grassroots memorials such as ghost bikes and other makeshift shrines commemorate death and loss, especially in times of trauma and social unrest (Margry and Sánchez-Carretero, 2022). Graffiti can contribute to contested collective memory (Grunow, 2019). Even the clothes people wear can act as memory (Hunt, 2014), symbolizing identity and group membership (Safdar et al., 2020).

#### Collective memory and high-tech external memory

High-tech external memory, by my definition, is that which does require a power source to operate (e.g., electricity). As seen in the historical context of low-tech external memory, the dazzling technology of the information age is merely a continuation of an ancient trajectory (Rumsey, 2016; Staley, 2014).^10^

##### Mass media

The technology of mass communication broadened collective memory with haste beginning in the early 20th century. Radio, movies, and television allowed rich new mediated experiences, and advertising, to be delivered to the masses. This emergence of mass media created *mass culture* (or popular culture), that could permeate a society. The broadcast era of the 20th century was one of shared consumption, “which provided both homogenization and stability to memory” (Mandolessi, 2023). Fictional narrative radio shows, music, television shows, and films became part of collective memory, as did broadcast news, and government propaganda (Black, 1977; Sproule, 1997). Bandura (2009) used social cognitive theory to explore how mass media can influence human thought, emotion, and behavior on the individual and collective levels.

Low-tech external memory never went away, with physical books, newspapers, and magazines remaining as part of mass media throughout the 20th century, eventually transforming to high-tech digital versions. Journalism has long played a key role in collective memory (Neiger, 2020; Zelizer and Tenenboim-Weinblatt, 2014). Kitch (2005) explored the role of magazine journalism in shaping collective narratives in the United States, “at a time when mass media have become a primary means by which most people understand the past” (Kitch, 2006). She further wrote: “as ‘the first draft of history', journalism is also the first draft of memory, a statement about what should be considered, in the future, as having mattered today” (Kitch, 2008).

##### Photography and video

Photography was another important development in external memory (Finley et al., 2018, pp. 113–116). Photos serve purposes for both individual and collective memory, such as material keepsakes, experience facilitation, identity formation, and communication (Fawns, 2020; van Dijck, 2007, pp. 98–121, van Dijck, 2008; Heersmink, 2023). Family photo albums construct and communicate family history and identity. They tend to underrepresent negative experiences in favor of an image of family unity and happiness (Frohlich, 2004, p. 39; Hirsch, 1981, p. 32). Personal photos can be powerful cues for shared memories and reminiscing.

At the public level, photos can serve documentation, artistic, or commercial purposes. Photos can imprint iconic moments of history in collective memory (Hariman and Lucaites, 2007). Examples include the view of Earth from the Moon, the haggard migrant mother of the Great Depression, the mushroom cloud of the atomic bomb, the man standing in front of tanks in Tiananmen Square, or boxer Muhammad Ali standing over his defeated opponent. Horrific photos of the Vietnam War, such as a girl burned in a napalm attack, likely shaped public opinion of the war in the Unites States (Sherer, 1989). As technology advanced, digital cameras and mobile phones allowed average citizens to photograph and film the authorities (sousveillance; Mann et al., 2003). This has resulted in collective action such as the protests following the public murder of George Floyd by police in Minneapolis in 2020, which was filmed by a bystander. In such cases, “citizen video replaces the work of professional journalists as ‘the first draft of history”' (Kitch, 2018). The capture and distribution of images and video makes injustice harder to deny or ignore. Many law enforcement agencies now require officers to use body-worn cameras (National Institute of Justice, 2022). In the age of social media, photographs and other images (e.g., image macro memes; Lankshear and Knobel, 2019) are widely distributed, sometimes going viral to become part of collective memory sometimes going viral to become part of collective memory (Berger and Milkman, 2012).

##### The internet age

The landscape of collective memory was further changed in the late 20th and early 21st century by the emergence of computers, the world wide web, smartphones, and social media. As opposed to the homogenization of collective memory in the broadcast era of mass media, these technologies have decentralized the creation and distribution of knowledge (the “memory of the multitude” as per Hoskins, 2018). Everyone can create and share content, and can find information in any niche. Collective memory, or popular culture (to the extent that there is such a thing anymore) has become kaleidoscopically fragmented. Mandolessi (2023) argued that digital technology has transformed contemporary mnemonic culture, replacing the static stable narratives of traditional archives with dynamic networked databases. “Transient, ephemeral, immediate and dispersed communities constituted through hyperconnectivity generate content that is constantly edited, re-edited, liked and altered, in a perpetual becoming.” Furthermore, algorithms and AI, while being based on the distributed activity of many users, obscure the process of information retrieval, and inch ever closer to the role of memory partners as in Wegner's transactive memory framework (Wegner et al., 1985).

In some ways, the wave of change in the internet age has revived older patterns. The parallels between oral tradition and internet technology were explored by Foley (2012) who argued that both mediums are similar to human thinking in being dynamic and progressing along pathways in a network rather than the linear progression that is characteristic of traditional static written text. Similarly, Pettitt (2012) proposed a Gutenberg Parenthesis model describing the movement from an oral culture to a print culture to a digital culture, with electronic media of the third restoring characteristics of the first, such as being more fluid and collaborative.

In an epilog to his book on oral traditions, Rubin (1995), argued that just as development of writing as a mental prosthesis transformed and expanded human thought and memory (see also Ong, 1982, Chapter 4), advances in computers and artificial intelligence would also do so. I note that the generative AI ChatGPT-4 assisted me in realizing this point, by creating a summary of Rubin's epilog. Thus, he was right. Orianne and Eustache (2023) argued that a progression of language and communication technology has enabled “autonomous social memory,” independent of living memories and social interactions, a sort of self-sustaining high-tech external memory.

Perhaps the most important site of collective memory in the internet age is Wikipedia (https://wikipedia.org), a free online encyclopedia that is collectively co-created and maintained by volunteers around the world. It is a repository of humanity's knowledge (collective high-tech external semantic memory), and also a forum for both contestation and consensus (Bruckman, 2022; Mesgari et al., 2015).

In addition to making an overwhelming amount of information available at all times, the internet age has transformed collective memory by the introduction of **social media**. Social media platforms (e.g., Facebook, Instagram, TikTok, Snapchat, X/Twitter, Pinterest, Reddit, Weibo) have enabled social connections and interactions unlike anything before in our species' history. We can connect with an unlimited number of friends, family, colleagues, and strangers all over the world. In this shared medium we can curate our life stories, and share our experiences in the form of text, pictures, and video. Sometimes we share individual past experiences. Sometimes we share a group memory, such as a photo with friends at an event, and we tag them in the photo, and they like the photo, and we may comment on it and reminisce together. The very interactions with others on social media are themselves experiences that can become shared memories (e.g., platforms like Facebook may remind us of a popular post we made some years ago).

What motivates sharing on social media? Wang (2020) developed a Purposes of Online Memory Sharing Scale, which measures four types of motives: self-related (e.g., self-expression, making a record for oneself), social-related (e.g., staying in touch, feeling close, networking), therapeutic-related (e.g., seeking advice), and directive-related (e.g., motivating or helping others). Stone et al. (2022) found that the primary motivations for sharing memories online were social, for both college students and older adults.

But there are downsides to social media. Rajaram (2024b) pointed out that the phenomena of social contagion and illusory truth (i.e., repetition begetting believability) can propagate collective false memories. Misinformation and conspiracy theories can be widely shared, and people may interact in online echo chambers, where their existing memories and views are amplified, whether accurate or not (Bernecker et al., 2021; Levy, 2023; Lewandowsky and van der Linden, 2021; Pennycook and Rand, 2018; see also confirmation bias, Klayman, 1995). There may be other consequences to social cognition (Sparrow and Chatman, 2013); for example, Echterhoff (2013) argued that the socially grounded construal of reality may be constrained for digital communication because users cannot infer each other's attitudes and feelings as well without nonverbal and physical cues. Additionally, the potential for addictive behavior is high (Levitin, 2014). For example, endless “doomscrolling” can result from the variable ratio reinforcement schedule of only periodically seeing an enjoyable piece of content (George et al., 2024).

Social media does offer a new forum for contestation of collective memory about the past. For example in 2015 the Chinese social media platform Weibo enabled public debates challenging official Chinese government narratives about historical events and figures (Liu, 2018). Compare this to the difficulty of recovering a suppressed collective memory of the Chinese Cultural Revolution (1966–1967), as described by Bonnin (2007).

Collective memory can lead to collective action, and social media can facilitate this, as in the Arab Spring anti-government protests in several Arab countries in the early 2010s (Wolfsfeld et al., 2013). But social media can also be used by politically motivated entities to influence public consciousness and collective memory (Birkner and Donk, 2020). For example, in 2016 the Russian government conducted a disinformation campaign, in part by using social media, to disrupt the U.S. election (Mueller, 2019). Culminating in 2017, Myanmar military personnel used fraudulent Facebook accounts to spread hate against a Muslim minority group, the Rohingya, inciting genocide (Mozur, 2018). It is also worth noting that large organizations such as businesses and national security agencies maintain large databases of information about people as a form of collective high-tech external memory (“big data”), which yields many possibilities for analytics but also contention over privacy (Christl, 2017; Price and Cohen, 2019), including the idea of the “right to be forgotten” (Rosen, 2012).

The design of objects and spaces can also carry the biases of a culture. Liao and Huebner (2021) defined *oppressive things* as “material artifacts and spatial environments that are in congruence with an oppressive system.” For example, automatic soap dispensers that only work for light-colored skin, or cameras calibrated to best capture light skin, reflect and perpetuate the racist notion that light skin is normal and desirable. The algorithms of social media are a high-tech example of how design can perpetuate bias. The concept of *universal design* aims to counteract such bias by making products and environments accessible to the widest range of people possible (Preiser and Smith, 2011).

Generative artificial intelligence has the ability to create convincing text, images, sound, and video from user prompts, based on learning from all of the human-created content of the internet. This kind of AI has only just exploded into widespread public availability in the early 2020s, and will almost surely further change the landscape of collective memory. Conceivably for the worse, because of the increased potential for creating disinformation and perhaps undermining public trust in legitimate sources of knowledge. But it will likely have unexpected consequences too, perhaps not all bad. In an early consideration of the possibilities of AI and memory, Hoskins (2024) argued that “the entanglements between humans and machines both enable and endanger human agency in the making and the remixing of individual and collective memory.” Is this a new class of tools we have made that will alter human affairs as radically as did written language, or the internet? Time will tell.

##### Death

Although information stored digitally can be endlessly copied across physical substrates, the continued accessibility of the contents of the internet is reliant on intentional upkeep and thus in some ways the storage is more fragile and ephemeral than low-tech external memory or even memory in the human brain (Borgman, 2000, pp. 202–205; Oliver and Harvey, 2016). Information posted to the internet may remain available forever, but it may also disappear without notice (Schloman, 2003). The Internet Archive (https://archive.org) stands as one attempt to preserve free access to the collective memory of the internet age for posterity.

Memorializing death is an ancient human tradition, with funeral rituals found across all existing cultures, and fossil evidence of human burial dating back over 70,000 years (Martinón-Torres et al., 2021). Low-tech external memory has long played a role in memorializing the dead, such as with headstones or kept artifacts of remembrance. Pitsillides (2016) thoughtfully explored the shift from physical material culture to digital presence in the context of death, noting that each of us in the internet age form digital footprints (traces of our thoughts, feelings, and actions online) and these can outlive us. This raises questions of one's digital legacy, or digital afterlife (Carroll and Romano, 2010).

As more of our lives and individual memories are encoded externally, the issue of curation arises. What is worth saving, and for whom? Digital traces of those who have died offer ongoing ways for the living to remember them, and perhaps even to interact with their echoes. Walter et al. (2012) noted that social media “can bring dying and grieving out of both the private and public realms and into the everyday life of social networks beyond the immediate family, and provide an audience for once private communications with the dead.” Conceivably, a customized large language model generative AI could learn from a lifetime of someone's written output and communications to create a chat bot simulacrum of that person that the living can interact with after death (Liu, 2023; Jee, 2022). Sisto (2020) discussed issues surrounding digital immortality, the role of social networks, digital inheritance, and high-tech funeral rites.

#### Collective memory and natural external memory

**Natural external memory** is a new addition to my concept of external memory. It is information stored in the natural world, excluding humans and their creations. When I originally defined technological external memory as “information stored in the inanimate environment” (Finley et al., 2018, p. 5), I briefly considered whether the living world (aside from other humans) might also serve as a form of memory. But, in an illustration of my limited perspective, the only example I could think of at the time was a rooster working as an alarm clock. Now, upon superimposing the lens of collective memory onto external memory, I realize that the natural world can indeed carry information from the past that can be retrieved by humans, individually or collectively. To some extent the natural world can be used by humans for encoding too, although this blurs the line with low-tech external memory. I have now revised my definition of technological external memory to be information stored in the *human-made* environment (no longer the inanimate environment), because parts of nature are also inanimate, in the biological sense, such as the rocks and the stars.

Indigenous human communities are those who inhabit a region for a very long time, developing close ancestral ties to the lands and natural resources of that area, and a distinct culture (United Nations, 2015). Many Indigenous Peoples have faced persecution, assimilation, displacement, disease, enslavement, or genocide by dominant colonizing groups who arrived much later. Thus, much unique collective memory—culture, language, knowledge, and beliefs—has been eroded or lost. But not all.

*Traditional Ecological Knowledge* is one term for the collection of knowledge, beliefs, and practices that Indigenous Peoples have acquired over time through direct contact with the environment, taught through oral tradition and in tribal schools and colleges. This collective memory could be seen as distributed across the minds of the individuals, their practices, and the natural world itself. There is much that the natural world can tell us of the past, if we have the knowledge to retrieve it. Just as in the Memory Symbiosis Framework for technological external memory (Figure 4), internal memory in the brain is needed to use natural external memory. This symbiosis is resonant with the Indigenous value of reciprocity with nature.

An edited volume called *The Land Has Memory* (Spruce and Thrasher, 2009) documented the establishment of the National Museum of the American Indian in Washington, DC, and does so in a way that also conveys Native values and worldview. Mother Earth gives us humans not just information, but life and lessons (e.g., take only what is needed). And in turn we must tend to her. Traditional Ecological Knowledge includes sophisticated land-management and agricultural techniques, such as controlled burning of prairies to facilitate grazing buffalo, to ensure the land we are tied to continues to give back. “So, just as each generation makes its mark on the land, the land inevitably makes its mark on us.” (p. xi).^11^ Kimmerer (2013) told the story of sweetgrass, which—contrary to the predictions of Western science—grows better when respectfully harvested than when left alone. This had been long known to the Native communities who harvest the grass to weave baskets and use in ceremonies.

“There is no place without a story,” wrote Johnpaul Jones in *The Land Has Memory* (p. 1), “Every plant, every animal, every rock and flowing spring carries a message. Native peoples of the Americas learned over thousands of years to listen to the messages, and we know every habitat. … We know the spirit of each living place.” This is Indigenous collective memory, tied to spiritual practices and to the environment, which to them are one and the same. Pascal (2023) explained, “indigenous moral and conceptual frameworks provide a different paradigm, one that imbues land and plants with personhood and agency.” Johnpaul Jones continued (p. 6): “Nature has something to teach us, not only through obviously animate things like plants and animals but also through rocks, mountains, rivers, and places large and small. These are all part of our spirit world.”

Such knowledge is in fact compatible with scientific collective memory (e.g., ecology, geography, hydrology, meteorology, astronomy), even if the two approaches speak in different terms (Johnson and Larsen, 2013; Starovoitov, 2021). Plants can tell us about the seasons, and more. Tree rings let us look into the past, showing burn histories and other climatic events. Footprints and scat in the soil tell us of animals who have come this way. The course of a river, and the sediment in its bed, tell us something of its history and perhaps its future. The mountains tell us of the deep geological memory of Earth. Scars in the land tell us where it was stripped for resources. The moon and the sun tell us the passage of time, and the stars tell us location. In some places there are culturally modified trees that show records of Indigenous human behavior such as bark stripping dating back hundreds of years (Mobley and Eldridge, 1992). Natural geographic features also have symbolic and spiritual meaning in Indigenous collective memory. For example, one of the Aboriginal Australian stories of the ancient coastline (Nunn and Reid, 2016) involved the seagull woman, Garnguur, who dragged her raft back and forth across a peninsula until it became an island. She was punishing her brother's negligence in watching her child. There are many other stories, but they are not mine to tell. More can be found if sought (e.g., Center for Humans and Nature, n.d.).

Even in non-Indigenous collective memory, people speak of *landmarks* that serve as spatial memory in the natural environment to help us navigate. Fernandez-Velasco and Spiers (2024) reviewed an incredible variety of Indigenous wayfinding techniques, many of which integrate environmental cues, specific tools, and a broader cultural system. Some techniques rely on encoding information into the natural environment, such as rock art (Zubieta, 2022; Whitley, 2022), which perhaps also qualifies as what I call low-tech external memory. Rock cairns as trail markers are another example. On the ocean, Polynesian Indigenous Peoples have long navigated using star constellations, wind directions, water currents, and sea birds. Memory and the natural world are tied together. Nunn (2018b) explained: “Geography and history in Aboriginal Australian societies were told as people moved along songlines, which were remembered routes across the land. Their memories were prompted by particular landforms.” Songlines have been described as “spatial stories woven from the autobiogeographical braiding of music and memory” (Roberts, 2023; see also James, 2013). They are similar to, but more elaborate than, the method of loci mnemonic (Yates, 1966).

In some Indigenous cultures, psychedelic drugs such as ayahuasca or peyote have been used as part of rituals to commune with the spiritual/natural world, for healing and/or access to knowledge (Luna, 2011). Boinnard (2024) related the teachings of a Peruvian medicine person, Arturo: “Healing and memory are very closely connected in the Indigenous world. You heal through connection, through *belonging*. The deeper the connection, the more sacred, so the sacred plants are all memory devices, helping us remember” (p. 98).

Indigenous collective memory is enmeshed with natural external memory, and embedded in a worldview of interrelatedness and reciprocity. Kimmerer (2013) explained: “We are bound in a covenant of reciprocity, a pact of mutual responsibility to sustain those who sustain us” (p. 382). Rituals and oral tradition sustain natural knowledge. The National Museum of the American Indian was built to nourish collective memory by way of oral histories, storytelling, and performances (Spruce and Thrasher, 2009). “Our elders say that ceremony is the way we can remember to remember. In the dance of the giveaway, remember that the earth is a gift that we must pass on, just as it came to us” (Kimmerer, 2013, p. 383).

### Collective memory and body memory

Here I will attempt to combine the framework of collective memory with the various systems of body memory I have proposed. This will involve some combination of viewing the systems through the lens of collective memory (e.g., cultural meanings), and treating the systems as themselves a kind of collective memory.

#### Collective memory and immunological memory

Immunological memory shows several interesting examples of what we might call collective memory. Public vaccination campaigns can seek to achieve **herd immunity**, whereby enough individuals in the population are vaccinated to protect a smaller number of people who cannot be vaccinated because they are too young or have health issues such as autoimmune diseases (Mallory et al., 2018). Herd immunity is clearly a form of collective immunological memory. If enough of the population's individuals have memory cells to protect against a pathogen, that pathogen can even be eradicated from the planet, as happened with smallpox, which the World Health Organization declared eradicated in 1980.

The beginning of an individual's immune system is also relevant here, showing intergenerational transfer. Before birth, immunoglobulin G (IgG) antibodies are transferred from the mother to the bloodstream of the fetus via the placenta (Jennewein et al., 2017; Palmeira et al., 2012). These antibodies exist because the mother's immune system had previously encountered and responded to antigens. Thus, vertically transferred **maternal immunity** can be enhanced through vaccination of the pregnant mother (Albrecht and Arck, 2020; Langel et al., 2022). The transfer of the IgG antibodies provides passive protection against common pathogens for the fetus during pregnancy, and for the newborn infant after birth. This transferred immunological memory is not indefinite; the maternal antibodies decay across the first year of life. The presence of the maternal antibodies interferes with the efficacy of some vaccinations, which aim to create memory cells; this is why some vaccinations (e.g., against measles, mumps, and rubella) are recommended to be administered at 12 months (Niewiesk, 2014; Centers for Disease Control Prevention, 2024). Thus, newborn humans possess some temporary immunological memory borrowed from the mother, increasing their chances of survival long enough to start developing their own adaptive immunity (memory B and T cells), which can be artificially boosted via vaccination.

In addition to the transfer of maternal antibodies, there is even some evidence that maternal memory T cells may be somehow transferred to the fetus during pregnancy and persist after birth (Kinder et al., 2017), although it is still unclear what affect these may have on the offspring's immunological memory. Furthermore, Kinder et al. (2017) argued that fetal cells transferred to the mother during pregnancy may improve the outcome of future pregnancies by the mother, especially with the same father; so the exchange of immunological memory is bidirectional.

After birth, transfer of maternal immunity can continue via breast milk. Breast milk, especially the colostrum produced in the first days after giving birth, contains a variety of antibodies that provide protection to the infant by coating their mucus membranes and digestive tract, where they temporarily protect against pathogens (Atyeo and Alter, 2021). This protection only lasts as long as the milk is present, so it is a sort of shorter-term transfer of immunological memory; that said, it is frequently reintroduced with feeding. This transfer can also be enhanced via vaccination of the nursing mother (Langel et al., 2022). Breast milk also contains memory B cells, although their effects are currently less well understood (Langel et al., 2022).

##### Microbiota

The intestines are where our body encounters many pathogens, but they are also host to many commensal bacteria (our microbiota) which help us digest nutrients and defend directly against pathogens alongside the immune system (Martín et al., 2013). Our immune system must develop a balance between adaptive immunity against pathogens and homeostasis with commensal microbiota (Eberl and Lochner, 2009). A critical time for such development is during birth. During vaginal birth, newborn infants are exposed to maternal vaginal and intestinal bacteria, which help seed the infant's intestinal microbiota, which in turn play an important role in boosting early immune system development. Research has found that infants delivered by c-section (cesarean delivery), who are not exposed to maternal bacteria in the same way, have less diverse intestinal microbiota, and are at increased risk of immune deficiencies (Coelho et al., 2021; Neu and Rushing, 2011). The infant gut microbiome is also seeded by maternal bacteria from breast milk and skin contact (Pannaraj et al., 2017). This is an interesting case of a form of long-term memory (transmission of information) directly from mother to child in the form of commensal bacteria. Antibodies in breast milk also help train the infant's immune system to be tolerant of commensal bacteria (Atyeo and Alter, 2021). Rotem and Rosenberg (2016) argued for the concept of the hologenome, the total DNA of a human and all of their symbiotic microorganisms (together considered a “holobiont”), which is responsive to the environment and thus a genetic vehicle for collective memory.

#### Collective memory and genetic memory

##### DNA

A human's DNA (their genome) is not just an individual record, but also a kind of record of the past before them. Of the 46 chromosomes comprising human DNA (23 pairs), half are inherited from the female parent, and half from the male parent. The specific DNA of an individual is put together (encoded) via the process of fertilization, which joins the 23 individual chromosomes of a sperm and egg, forming a zygote, which can develop into a blastocyst, embryo, fetus, and infant. In this way, an individual's DNA forms a partial record of their parents' DNA. And of course, each parent's DNA was combined from their parents before them, and so on back through the history of our species and beyond, to the earliest primordial origin of DNA on this planet. Such genetic memory does not of course contain the neurological memories of one's forebears, nor any other acquired characteristics. But rather what is inherited are phenotypical traits, the observable characteristics of an organism that arise from the interaction of its genetics and the environment. Heritable phenotypes in humans include physical traits such as size, shape, and pigmentation, and mental traits such as temperament (Saudino, 2005), personality (Jang et al., 1996), and general intelligence (Plomin and Deary, 2015). Predisposition toward various physical illnesses (Polubriaginof et al., 2018) and mental disorders (Pettersson et al., 2019) are also heritable to some extent. The actual occurrence of mental disorders can depend on an interaction between genetic predisposition and environmental stressors (the diathesis-stress model).

The totality of all human DNA is the genetic memory of the species. It is collective memory in both breadth (variance) and depth (into the past). Although the average amount of genetic variation between two humans is only about 0.1% (meaning we all have 99.9% of our genes in common), that 0.1% accounts for all the vast diversity in human phenotypes (National Institutes of Health, 2007).

Humans also have mitochondrial DNA (mtDNA), contained in the mitochondria organelles of our cell nuclei. This is a separate line of genetic memory, and is inherited from the mother's side. Such mtDNA can be used to trace a maternal line of descent back to a matrilineal most recent common ancestor to all modern humans, a woman estimated to have lived between 99,000 and 148,000 years ago in East Africa (Behar et al., 2008; Poznik et al., 2013). The very presence of mitochondria in our cell nuclei is a record of ancient endosymbiosis, when one single-celled organism engulfed another, creating the first eukaryotic cells some 2.2 billion years ago (Cohen and Kodner, 2022).

Modern genetic analysis techniques have allowed us to plumb the depths of human genetic memory. For example, analyzing autosomal DNA (non-sex chromosomes), Y-chromosome DNA, and mtDNA, has shed light on the timing and locations of human migration over the past 200,000 years, both within our original continent of Africa and beyond (De Knijff, 2010; Underhill and Kivisild, 2007). Genetic analysis has also become available to consumers, yielding a field of personal genomics services, such as 23andMe and AncestryDNA, which allow people to submit saliva samples (containing DNA) to explore their own genealogy, identifying relatives and likely locations of ancestors (based on haplogroups).

##### Evolution

The process of evolution by natural selection can itself be considering a kind of learning process that unfolds over eons, yielding the genomes of all organisms alive today, as well as the fossil record (a kind of planetary memory of life; Schopf, 1999). Evolution is driven by variation in organisms, due to genetic mutation and recombination (e.g., sexual reproduction). Genetic variants persist when they yield phenotypes that are advantageous to survival and reproduction in their given time and place. Otherwise, genetic variants die out. In this way, although it is a blind process with no agency or goals itself, evolution learns.^12^ The products (or memory) of this learning are not only the species themselves, but also certain behaviors and predispositions. For example, there are hard-wired reflexes such as automatically withdrawing a limb from a painful stimulus, or blinking in response to a puff of air. These reflexive responses are present from birth in many animals. They did not have to be learned in the lifetime of an individual (ontogeny); instead, the learning happened through evolution by natural selection (phylogeny; Rapoport, 1984; Skinner, 1984). Ancestors with such tendencies were more likely to survive and pass on their genetics than ancestors without such tendencies. All of classical conditioning is reliant on such preexisting associations between unconditioned stimuli and unconditioned responses.

In addition to simple reflexes, some organisms also inherit more complex behavior, such as weaverbirds who instinctually build complex nests, even if raised in isolation (Collias and Collias, 1964), and greylag geese who show the fixed action pattern of rolling a displaced egg back into the nest (Lorenz and Tinbergen, 1970). Organisms can also inherit predispositions toward developing certain behaviors in species-typical environmental contexts (e.g., migration, group dynamics; Blumberg, 2017; Kenrick et al., 2006).

##### Epigenetics

Recall that epigenetics is the changing of how genes are expressed in an individual's DNA, by binding of a methyl group molecule to the DNA or modification of histone molecules the DNA is wrapped around. These epigenetic markers occur for a variety of reasons (e.g., normal development, environmental stress). They are copied when cells divide in mitosis. However, evidence from mice suggested that for mammals the methyl epigenetic markers are generally stripped away during the process of meiosis, which creates the haploid sex cells (i.e., gametes: egg or sperm), and stripped away at the time of fertilization when egg and sperm combine into a zygote (Monk et al., 1987).

Because epigenetic markers were thought to be stripped away (reprogrammed) during meiosis and fertilization, it followed that there was no way that they could be passed on to offspring. However, later research with mice has shown that some locations of the DNA can in fact retain methyl epigenetic markers across meiosis and fertilization (Daxinger and Whitelaw, 2012). So it turns out that some epigenetic markers on the DNA of gametes could in principle be transmitted across generations. This has been demonstrated in mice. For example, Dias and Ressler (2014) classically conditioned mice to fear the smell of cherries, which they normally are attracted to. They found that the next two generations of mice also feared the cherry smell, and this even held true when they were raised apart from their biological parents or conceived by *in vitro* fertilization. The fear response had apparently been transmitted via an epigenetic marker on the sperm of the conditioned male mice, which led to altered pathways of olfactory nerves.

What about in humans? The issue is complicated (Ghai and Kader, 2022), in part because it can be difficult to rule out social and environmental vectors of transmission as opposed to direct transmission via the epigenome of gametes (Lehrner and Yehuda, 2018; Nagy and Turecki, 2015). But there have been some epidemiological studies suggesting **transgenerational inheritance of acquired characteristics**, for which epigenetics is a possible mechanism. Most of these studies focus on the impact of hardships during pregnancy. For example, Schmitz and Duque (2022) found that people who were in the womb during the worst of the US Great Depression in the 1930s later showed epigenetic markers of accelerated aging. Research has also documented health and disease outcomes in multiple generations following exposure to famine at time of conception or pregnancy (Painter et al., 2008), and even identified epigenetic markers involved (Tobi et al., 2018). There has also been evidence that experiencing trauma before pregnancy can lead to epigenetic changes in later offspring. For example, children of Holocaust survivors have shown altered epigenetics along with a higher predisposition for developing PTSD (Yehuda and Lehrner, 2018). Research is ongoing regarding the extent to which effects of environmental factors can be transmitted across human generations via epigenetics (Fallet et al., 2023). However, it does seem plausible.

Whereas genetic memory (DNA) provides a molecular record of the deep history of Darwinian evolution, *epigenetic* memory may provide a Lamarckian back-channel of more limited scope, recording echoes of experience across several generations. Note that this kind of transgenerational epigenetic memory is at the level of altered gene expression, not the level of the potentiated patterns of neuron activation that comprise long-term memory in the brain. So people do not inherit, say, episodic memories from their parents; but rather, they may inherit genetic predispositions influenced by their parents' experiences.

#### Collective memory and skin

The range and geographical distribution of skin pigmentation is a kind of collective memory of the migration and evolution of our species over the millennia, and the interaction of genetic, environmental, and cultural variables (Jablonski, 2021a). The idea of **race** as a classification of humans, although nominally based on skin color, is a social construct (Cheng, 2008; Jablonski, 2021b). Thus, the idea of race is part of the collective memory of a society, and one that has consequences in the many effects of racism, which include everything from the suppression of civil rights, education, economic opportunity, and medical treatment (Williams, 1999) to slavery and genocide. These consequences can become topics of contestation in collective memory, for example in struggles to publicly acknowledge long-silenced histories of racial violence (Whitlinger, 2015). Racial and ethnic identities can comprise communities that have their own collective memories (Bindas, 2010). As one example, Black vs. White Americans showed different prevalences of flashbulb memories for the assassinations of civil rights leaders, and different ratings of the importance of those events (Berntsen, 2017).

Scars on the skin can be markers of collective past trauma. Mwambari and Sibomana (2023) wrote of post-genocide Rwanda: “Bodily scars play powerful and complex roles in memory conversations; they communicate trauma and keep memories of the mass violence vivid in public and private realms.” In Chile, a former political prisoner visited a detention center turned into a historical memory site and commented: “I think memory sites are the same as the scars in your body, which remind you that the past was true” (Morales et al., 2022). There have been cultures, such as the Lango people in Uganda, in which people make cuts that intentionally scar their own body to mark the number of enemies killed (Lagercrantz, 1973); this acts as an individual body memory, but also communicates prowess in a way understood by the community. These examples show the embodiment of collective memory, a topic further discussed by Narváez (2006, 2012).

#### Collective memory and hair

Hair style can be a salient symbol of group identity (Synnott, 1987). For example, hair has long been an important part of identity for the Black community (Lashley, 2020; Thompson, 2008). When African people were enslaved and brought to North America, they were stripped of their language, culture, and hair. As deliberate acts of dehumanization, their hair was shaved and they were forbidden haircare products (Byrd and Tharps, 2014). Nevertheless, enslaved African American people developed ways to style and care for their hair with whatever means they could, for example styling braids or cornrows. Even after emancipation, Black hair texture and style were seen as inferior by White Americans, and targeted for discrimination; thus there was pressure to assimilate by straightening curly hair to conform to White beauty standards (Varnado, 2023). Around the time of the Civil Rights movement in the 1960s, natural hair styles (e.g., afro) began to gain popularity as expressions of pride, and in defiance of negative stereotypes (Patton, 2006). In 2022, the U.S. passed the CROWN (Create a Respectful and Open Workplace for Natural Hair) Act, aimed at protecting against workplace discrimination against African American hairstyles. There is even contention about representation of authentic Black hair styles in video games (Williams, 2019). Regardless of style, Black hair continues to be an important part of the community's culture and collective memory.

For Native American people too, attempts to erase their culture included cutting children's hair as part of forced assimilation at boarding schools (Adams, 1995, pp. 100–102), and a 1902 order for adult men to cut their hair or else receive no food rations (Onion, 2013). Societal attitudes have also cast negative judgments on women's body hair in general, demanding its removal (Basow and Braman, 1998; Fahs, 2022).

#### Collective memory and body modification

Tattoos can convey not just individual information, but also group information. For example, for the Māori people, the tā moko tattoos are a sign of cultural identity, and different tattoos can indicate social rank (Simmons, 2004). Some descendants of Holocaust survivors have replicated the concentration camp tattoos of their parent or grandparent as a memorial practice (Bloch, 2024). Tattoos associated with prisons and gangs can convey meanings that vary regionally, for example indicating gang affiliation or amount of time served. The Yakuza organized crime syndicate of Japan uses extensive body tattooing as markers of loyalty, leading to a broader cultural stigma against tattoos in Japan.

There have been many other cultural practices of body modification that convey group membership, such as piercings (Weiler et al., 2024) and scarification (Lagercrantz, 1973). Male circumcision in some cultures is a rite of passage (Toksoy and Taşitman, 2015). Other culturally influenced body modifications include skin lightening (Faria, 2022) and cosmetic surgery (Bonell et al., 2021). In terms of makeup, there has been movement toward cosmetics that are inclusive of all skin tones over the years, fueled in part by online communities (Garschi, 2024).

#### Collective memory and nails

Decoration of fingernails is a tradition in many cultures, and dates back thousands of years (Shapiro, 2014). It is generally practiced more by women than men, although this varies across cultures. Long or decorated nails are not practical for those who labor with their hands, so such decoration can be a symbol of status or wealth (DeMello, 2007). The meaning of certain styles varies across communities (Fetto, 2021).

#### Collective memory and bones and teeth

The condition of one's teeth can convey social information. In North America, the prevailing aesthetic ideal is to have straight white teeth, and a great deal of money is spent on orthodontics and tooth whitening to attain this. Khalid and Quiñonez (2015) pointed out how this reinforces class differences, as people with lower income are excluded.

#### Collective memory and muscles and movement

Dancing is a widespread and ancient human behavior, usually existing in a cultural context (Hannay et al., 2024). Dance often consists of ritualized movements coordinated across multiple people, and can foster social ties and group identity (Fink et al., 2021). Styles of dance are transmitted across generations as collective memory, for example as part of rituals and celebrations. Dance can also be an integral part of oral tradition (Scheub, 1985). The act of dancing with partners has been studied as a form of distributed cognition and collaborative memory (Stevens et al., 2019). Tewes and Fuchs (2018) introduced a special issue exploring the idea of body memory as habits and embodied practices in the context of cultural norms and styles of expression. One could argue that dance is nothing more than procedural memory in the brain. However, the pattern of muscle strength and the ease with which one performs a given dance style reflect their history of movement.

#### Collective memory and voice

Language is absolutely essential to the human experience and is the bedrock of collective memory. It is what enables oral tradition, which was the only way to pass on knowledge across generations for most of human existence. The language spoken by a group is part of the fabric of its collective memory. To some extent, language can shape the thoughts of a people (Boroditsky, 2001, 2011). There are currently a great many human languages—over 7,000 as of this writing, but with over 3,000 of those in danger of extinction (Ethnologue, n.d.). Eliminating the language of a people, for example by forbidding its teaching, is a way of destroying a culture, and was part of the original definition of genocide (Lemkin, 1943). Even regional accents and dialectical differences within the same language can be markers of group identity. Code-switching is when someone changes their language or way of speaking to fit a social context.

Singing, like dancing, is another ubiquitous human behavior. Singing can facilitate social bonding (Kreutz, 2014), and serves as communication for emotional expression and group membership (Welch, 2005). Songs play an important role in oral tradition (Rubin, 1995; Scheub, 1985). Music is a common and powerful source of nostalgia, especially when tied to culture (Garrido and Davidson, 2019).

#### Collective memory and digestion and excretion

As a response to the COVID pandemic in the early 2020s, epidemiologists have been able to test the wastewater (sewage) of a community to monitor the circulation of viral pathogens in the population (Ciannella et al., 2023). The combined fecal matter of a community thus serves as collective memory of infection levels.

#### Collective memory and blood

Blood donation is a life-saving practice of sharing one's blood with the community. Donated blood can be transfused to patients to help them recover from surgery, trauma, or illness. Compatibility of blood type matters, so having a heterogenous blood supply is important. Because blood can carry disease and infect recipients (a kind of memory transfer), donations need to be tested, for example to detect antibodies to Hepatitis C and HIV. Community values and beliefs can influence blood donation practices (Smith et al., 2011).

#### Collective memory and reproductive systems

Sex is heavily influenced by culture. A given community's collective memory will include norms and beliefs that influence sexual behavior. The birth rate of a community and the incidence of sexually transmitted infections both reflect the frequency of unprotected sexual intercourse, which is in turn influenced by education and attitudes about condoms and birth control (Lindberg and Maddow-Zimet, 2012; Zuilkowski and Jukes, 2012). The collective memory of the internet provides means for people to access both valid and invalid information about sex and reproduction. Access to reproductive health, such as birth control, abortion, vasectomy, and infertility treatment, also varies across communities. In some cultures, the erroneous belief that a woman must show an intact hymen to prove virginity has driven the practice of hymenoplasty (Wynn and Hassanein, 2017). The mean age of menarche is lower in more developed countries (Morabia et al., 1998), likely due to nutrition.

#### Collective memory and fat

Attitudes about body fat vary across cultures (Rodin, 1993). In some cultures, fat is a symbol of wealth, power, or fertility (Musaiger et al., 2004). In others, thinness is idealized (Swami et al., 2007). Thus, the social meaning of fat is part of a group's collective memory.

Some research has suggested that obesity can be socially contagious, in that it can be observed to spread through social networks over time. Several reasons have been proposed, such as conformity to a social weight norm influencing eating behavior and/or physical activity (Huang et al., 2016; Mathieu-Bolh, 2020).

#### Collective memory and lungs

People in a group share the air they breathe. Secondhand smoke comes from someone smoking tobacco, and is inhaled by a second person who is not smoking. Secondhand smoke increases the risk of heart disease (Barnoya and Glantz, 2005) and lung disease (Su et al., 2024). The lung condition of a group of people who spend extended time together (e.g., a family) is a kind of collective memory that is influenced by the smoking behavior of even just one individual.

#### Collective memory and body-based numerical representation

Although finger counting is a universal method of offloading working memory, the exact way it is done varies across cultures. Bender and Beller (2012) reviewed an impressive diversity of counting strategies. For example, many Western European counting systems start on the left hand (e.g., starting with the thumb), while the Iranian system starts with the right hand's pinky finger. The Oksapmin people in Papua New Guinea use a counting system incorporating 27 points across the upper body (e.g., wrists, elbows, shoulders, ears). There are even different number bases used in different systems. While many systems use base 5 (quinary) or base 10 (decimal), there are also some that use base 20 (vigesimal) by counting all fingers and toes. The authors developed a typology of systems and discussed the variance in ease of learning and cognitive load. Different systems have different tradeoffs between memory representations in the brain vs. the body.

## Discussion

I have presented a series of taxonomies of human memory. I began with the prevailing taxonomy of memory systems in the brain as traditionally studied by cognitive psychologists (Figures 1, 2). This includes sensory memory, short-term/working memory, and long-term memory, with the latter being further subdivided into explicit (including episodic and semantic) and implicit. I followed this prevailing taxonomy with three expansions. The first expansion added the idea of external memory, which is information available to an individual outside of themselves (Figure 3). I differentiated between social and technological external memory (including low-tech and high-tech) and I discussed the symbiosis of internal and external memory (Figure 4). The second expansion introduced memory in the human body outside of the brain (Figure 5). The biggest two systems I detailed were the immune system and genetics (including DNA and epigenetics), followed by a variety of others: skin, hair, body modification, nails, bones and teeth, muscles and movement, voice, digestion and excretion, blood, reproductive systems, fat, lungs, and body-based numerical representation. Finally, the third expansion introduced the superordinate distinction between individual and collective memory, and added natural external memory (Figure 6). I discussed views of collective memory and what it consists of, and then I explored how collective memory relates to or recasts the entire rest of the taxonomy, including the systems of the brain, the systems of external memory, and the systems of the rest of the body. Throughout, I have highlighted parallels and differences between the variety of human memory systems, as well as ways in which they work together.

This has obviously been a sprawling endeavor that cuts across many different fields of study. It is no coincidence that the idea originated in an interdisciplinary undergraduate honors course I created, *Memory and the Human Experience*, at a small liberal arts college, which made for an excellent crucible of ideas (Finley, 2017). The course explored the theme of memory in human life by integrating perspectives from the sciences, arts, and humanities.^13^ Why do I think that helped? The “beginner's mindset” is a concept from Zen Buddhism (Suzuki, 1970), that encourages us to adopt an attitude of openness and eagerness, and to abandon the preconceptions that come with the development of expertise. This enables a fresh approach to even complex topics, and can facilitate new insights and frameworks. I hope that I have achieved something like that here.

### Limitations

Despite my level best, there may be additional systems I have not considered. Those are not included in this manuscript. The endocrine system is one possibility (Le Tissier et al., 2017). Of the systems I have included, I may have left out important examples. And there are some places where the boundaries between systems are unclear. For example, simple alterations to the natural environment (e.g., stone cairns) seem to straddle low-tech external memory and natural external memory. The notion of social external memory makes sense from the perspective of an individual, but when adding the superordinate category of collective memory alongside individual memory, one could argue that social external memory becomes redundant, except perhaps to capture social interactions. But I do think that oral tradition fits nicely there. The distinction between social and technological external memory will continue to erode as artificial intelligence advances. It is not clear exactly how to constrain the overall idea of collective memory, although I am far from the first to struggle with that. At least it is true that the edges between proposed systems are also blurry even for memory in the human brain (Buckner, 2007), and the expanded taxonomies do have the advantage of relying on distinctly separate physical substrates.

That said, the issue of how to *measure* memory across such a variety of systems is a difficult one. Measurement techniques may be impractical and would vary widely, hindering standardization and comparison across systems. For example, measurement would range across: genetic sequencing; blood tests for immune and blood memory; visual inspection of obvious features such as hair, skin, and nails; x-rays for imaging bones and teeth; auditory analysis of voice; reading and comprehending written words (which requires knowledge of the language and cultural context); interpreting visual art; recording oral history; and using the appropriate software and hardware to access high-tech external memory. Reconciling a multiplicity of measurement techniques is a challenge even just within long-term memory in the brain (Brady et al., 2023; Otani and Schwartz, 2019). Furthermore, in many cases the information retrieved from a memory system is ambiguous; for example, several possible pasts could all yield a similar scar on the skin, and both infection and vaccination could yield the same memory cells in the immune system. But such under-determination is a characteristic of memory in the brain too; the same recollection could be a product of true original experience, misinformation, false memory, schema inferences, or some combination thereof.

There are other ways to slice up human memory into different concepts. For example, Roediger et al. (2017) gave one such typology. Some concepts of human memory—such as autobiographical memory, repisodic memory, prospective memory, event memory—do not even fit nicely into the prevailing taxonomy of the brain. Prospective memory in particular (i.e., remembering to do something in the future; McDaniel and Einstein, 2007) is a regrettable omission because it is such a common part of everyday life; but it is perhaps more of a purpose or process than a system in its own right. That said, Cubelli (2010) proposed a different taxonomy of long-term memory, dividing it into reproductive systems (procedural and semantic) vs. reconstructive systems (episodic and prospective), instead of the usual division of explicit vs. implicit. There are likely more phenomena that do not fit well into the expanded taxonomies. However, Buckner (2007) made a keen observation that is relevant here: “Use of memory in everyday life certainly does not respect boundaries between memory systems but rather flexibly draws upon multiple available systems to attain goals.” (p. 363).

Finally, as mentioned in the Introduction, the very creation of memory taxonomies implicitly endorses structuralism and the storehouse metaphor of memory, downplaying functionalism and the correspondence metaphor (Koriat and Goldsmith, 1996). This means less focus on the dynamic processes of memory, the accuracy of retrieved information relative to the actual past, and interactions between different elements (e.g., contextual effects, transfer-appropriate processing) or even between different systems. These are areas for further development.

### Lessons

Roediger (2003), a mentor of mine, warned against stretching the term “memory” too far (p. 14). I went ahead and did so anyway. To what end? I return to the reflection by Young (1965): “The technique of pushing the analysis of the system as far as possible… seems to have brought increasing clarification.” Has clarification increased? Or perhaps instead the following timeless quote is more apt:

We have not succeeded in answering all our problems… The answers we have found have only served to raise a whole set of new questions. In some ways we feel that we are as confused as ever, but we think we are confused on a higher level, and about more important things. (Kelley, 1951, p. 2)

Despite the limitations, I think the expanded taxonomies do a serviceable job of juxtaposing many views of human memory, and revealing new insights. The idea of natural external memory was an important addition that arose from the extended taxonomies. I was also forced to revise several definitions: internal memory, external memory, and technological external memory. Whereas the distinction between internal and external memory we made in Finley et al. (2018) was in reference to the individual's brain, in order to accommodate other forms of body memory I had to change the reference to simply the individual. And the addition of natural external memory forced me to change the definition of technological external memory from referring to the inanimate environment to the human-made environment.

My background as a cognitive psychologist drove my definition of memory as the transmission of information across time, which is what I based these extended taxonomies on. However, many of the same distinctions could be arrived at by using a more behaviorist view, defining learning as a change in behavior resulting from experience. Roediger (2003) also highlighted an 1870 address to the Imperial Academy of Science in Vienna by Ewald Hering, titled “On Memory as a Function of Organized Matter” which Edgell (1924) recounted as follows:

Memory is not merely a term used to denote a given collection of facts, but is used as an explanatory principle. Advance of the race, reproduction of one generation by another, material growth, increase in skill, recollection of the past, they are all explained by that function which is universal in organized matter: memory. If one tries to generalize these very diverse facts and regard them as a class one may term them each and all “after-effects of stimulation.” (Edgell, 1924, p. 7)

Thus, both a behavioral view and a cognitive view are compatible with, or at least relevant to, the expanded taxonomies.

One overall lesson from the expanded taxonomies is this: by considering human memory across different underlying substrates and different levels of analysis, we can see how the various systems are sometimes parallel, sometimes complementary, and sometimes in opposition. There are multiple memory concepts that appear and recur across the systems, including in no particular order: encoding, storage, retrieval, priming, savings in relearning, consolidation, capacity, duration, forgetting, amnesia, malleability, errors, false memory, reconstruction vs. reproduction, transfer, retrieval practice, spacing, interference, repression (and lack thereof), symbiosis, reciprocity, contention, embodiment, voluntariness, modalities, bias, directive function (e.g., prospective), schemas, strategies, types of information (e.g., episodic, semantic, repisodic, procedural), evolutionary origins, and so on. There are likely even more parallels and distinctions. Analogies and metaphors have driven theory before (Roediger, 1980).

#### Directions for future research

The expanded taxonomies are descriptive rather than explanatory (Willingham and Goedert, 2001), so although they can serve to inspire theory, they cannot serve as theories themselves, and thus do not directly yield testable predictions. Nevertheless, they suggest directions for future research by identifying common characteristics and processes across different memory systems. I have just listed a number of cross-cutting concepts in the previous section. Those concepts originated in the study of neurological memory, but could be fruitfully applied to the other systems, despite the challenges in measurement. I will list here a few examples.

It should be possible to more formally estimate the general capacity and duration of the various forms of memory, along with other characteristics. In Finley et al. (2018) we proposed important dimensions and unanswered questions about external memory (pp. 155–173) along with relevant research methods (pp. 144–150).

Across systems, different encoding strategies may yield different strengths and durations of memory. For example, this could apply to the protections afforded by different kinds of vaccines (Pitisuttithum et al., 2021) or methods of vaccine administration (Monto et al., 2009).

The spacing effect occurs for neurological memory, immunological memory, tattoos, and annual observances for collective memory. The same principle might or might not hold for other systems. For natural external memory, there may be spacing effects in terms of distance between landmarks in songlines, a cultural memory structure combining spatial, temporal, musical, and spiritual elements.

Something like priming is already at work in some high-tech external memory: autocomplete is a feature that predicts a user's input while they are typing, and it can be based on both recency and frequency of previous input by that individual and/or a collective. In some file system views, frequently and/or recently used files are listed more prominently. The principle of priming may be useful in other ways and in other systems too.

Modalities have been well studied for individual neurological memory. How might that concept apply to other systems, and at the collective level? Certainly for natural external memory, information from the world could be delivered through all of the human senses.

With regard to retrieval processes across systems, neurological memory is more reconstructive and external memory is more reproductive, yet both contribute to collective memory. Knowledge of the strengths and weaknesses of theses memory systems may enable those with agendas to shape cultural narratives.

The concept of bias cuts across systems: bias in what is encoded, stored, and retrieved, at the individual and the collective level. This deserves investigation.

The concept of voluntary vs. involuntary memory retrieval is worth exploring more. I identified the autoimmune skin disease psoriasis as an example of involuntary memory, but there must be more. Online advertisements and suggestions could be considered involuntary high-tech external memory. Obsessive-compulsive disorder symptoms such as compulsive checking could be recast as repeated involuntary retrieval of information from the environment (external memory). But how does the concept of voluntariness apply at the collective level, where this is no single agent?

False memory is a potential for perhaps all of the systems, given the ambiguity of information retrieval. This carries forensic and clinical implications. Bruises on the skin may appear to be records of an accidental injury when they are actually records of an assault, or vice versa. Biomarkers from blood, hair, nail, or fecal samples may appear to indicate one illness when they are actually due to another. Even with external memory, false memory could result from human error or fraud at encoding, mislabeling or tampering during storage, or even system errors delivering the wrong document at retrieval. Generative AI has enabled encoding of more and more believable false information in high-tech external memory (e.g., fake pictures and videos), and “retrieval” of incorrect but plausible-looking information, a phenomenon known as hallucination in large language models like ChatGPT (Huang et al., 2024). Perhaps some amount of ambiguity is inherent to any memory system, and thus judicious use of additional decision processes are required when using information from the past. The framework of signal detection theory has been applied to study and evaluate a wide range of such decisions, from individual memory decisions in the brain to medical diagnosis decisions based on external memory such as radiographs (Swets and Green, 1978).

The concept of different kinds or purposes of long-term neurological memory (e.g., episodic, semantic, procedural, prospective) has been fruitfully applied to technological external memory (Finley et al., 2018; Finley and Naaz, 2023). In this manuscript I have speculatively applied those terms to other memory systems as well (e.g., skin, tattoos), but more exploration is needed.

Finally, a *complex systems* perspective (Bar-Yam, 2002; Ladyman et al., 2013; Newman, 2011) could be applied to the taxonomies to study the ways the multiple systems interact with each other. Heersmink (2023) explored interactions between collective memory, individual neurological memory, and artifacts (technological external memory). Gaddy (2023) explored the idea of “demographic memory” that combines social and biological sciences, emphasizing and quantifying the role of survivorship. We previously explored the interplay between internal memory in the brain and technological external memory in Finley et al. (2018, pp. 49–72, 140–142; see also Figure 4).^10^ In the previous section on Collective Memory and Long-term Memory: Processes, I explored parallels, differences, and interactions between the individual vs. collective levels, including spacing, repression, consolidation, forgetting, and the availability heuristic. I also noted that immunological memory impairs neurological memory when fighting infection. But there is much more still to explore regarding processes and interactions among all the various subsystems. For example, external memory and genetic memory are more similar to each other than to neurological memory in terms of precision and self-correction of errors. If each of the memory systems has vulnerabilities (e.g., forgetting, false memory), to what extent does the combination of the systems together amplify or dampen the risks of error? What self-organizing patterns emerge from the variegated patchwork of memory systems that comprise human experience? How will the interplay between internal and external memory continue to evolve, at both the individual and collective levels? These questions and more await answers.

### Big picture

Here I will conclude with several bigger picture issues related to the expanded memory taxonomies.

#### Nature vs. nurture

The classic issue of nature vs. nurture is about the extent to which the characteristics and behaviors of an organism are due to evolution and genetics (nature) vs. environment and learning (nurture). In the section on Collective Memory and Genetic Memory, I argued that evolution itself is a kind of learning process that yields well-adapted phenotypes in addition to innate stimulus-response associations, some instinctive behaviors, and predispositions toward developing adaptive behaviors. The latter is a combination of nature and nurture (Blumberg, 2017; Kenrick et al., 2006; Burnham and Phelan, 2001), with examples including migration, navigation, kin altruism, reproductive strategies, associative bias in fear conditioning (Garcia and Koelling, 1966), predisposition toward fear of ancient ancestral threats (e.g., snakes; Prokop, 2016), and language learning in humans (Pinker, 1994).

Furthermore, evolution has played a driving role in the emergence of most of the memory systems I have covered. About memory in the brain, Westermann et al. (2015) wrote: “Why do we form memory?—because innate response patterns do not suffice to warrant survival of a species if the environment of the organism changes at a rate much faster than its reproductive cycle.” Tulving (1995) similarly wrote: “Memory is a gift of nature, the ability of living organisms to retain and to utilize acquired information or knowledge.” For an entire volume on the adaptiveness of neurological memory, see Schwartz et al. (2013). Most of the biological memory systems, all in the Internal category (Figure 6), are clearly products of evolution. Brains evolved to adaptively guide behavior, the immune system evolved to adaptively fight infection, and genetics is the very code underlying evolution. Most of the body memory systems are also products of evolution, even if their capacity to transmit information over time is a byproduct of their biological function (e.g., scars forming on skin due to healing). However, body modification and body-based numerical representation are products of culture (nurture). And even for the biological memory systems that arose through evolution, the content they learn is from nurture—personal experience, influenced by environment and culture.

External memory on the whole is entirely nurture, except to say that it is in human nature to build tools and extend ourselves into the environment. Adding the superordinate category of collective memory complicates matters. On the one hand, collective memory appears to be completely nurture-based: cultural transmission of information, contestation, and consensus. Humans must learn the contents of collective memory through experience and education. On the other hand, the individual biological memory systems underlying such learning did arise through nature. And the very proclivity for such learning, and indeed the capacity for social learning of behaviors (Whiten et al., 2004), is an innate tendency in humans. Furthermore, there are some universals across human cultures, such as facial expressions of basic emotions (Hwang and Matsumoto, 2015), the centrality of language, and behavioral predispositions such as ingroup bias and fear tendencies (Kenrick et al., 2006). Epigenetics, as a possible vector of information transmission across generations, lies at the intersection of nature and nurture. Overall, the memory systems in the expanded taxonomies vary in the extent to which they are products of nature vs. nurture, and the extent to which their operation is a process of nature vs. nurture. On balance, I would say that learning is nurture, influenced by nature.

#### Memes

This manuscript covers the various human memory systems in which information is encoded, stored, and retrieved. What if the focus of analysis was not on the systems, but on the information itself? That was the original idea of **memes**, a term coined by Dawkins (1976) to mean the units of cultural replication and transmission, by analogy to genes as the units of biological replication and transmission. Such a notion dates back to Darwin's time, with Huxley (1880) writing: “The struggle for existence holds as much in the intellectual as in the physical world. A theory is a species of thinking, and its right to exist is coextensive with its power of resisting extinction by its rivals.”

The idea of memes is particularly applicable to collective memory, as it concerns how information persists and evolves. Memes propagate themselves across individual brains and repositories of external memory. The particular physical substrates do not matter so much. The same meme could exist in a brain, a book, or a computer. This speaks to the overall idea of memory as a function of organized matter.

Whereas biological evolution is based on genes and is Darwinian (differential survival among spontaneous variations), cultural evolution is based on memes and can be Lamarckian (inheritance of acquired characteristics) in that memories can be modified and then passed on, just as in Bartlett's (1932) demonstration of the distortions that can occur when messages are passed along by a series of people. There is debate on this, however, with Mesoudi (2011) claiming that cultural evolution is more Blackmore (1999) claiming the entire analogy is misguided. Despite the efforts of some scholars (e.g., Distin, 2005; Lake, 1998; Shifman, 2013), the study of memes—memetics—has apparently not yet coalesced into a fruitful field. But I think the ideas of memetic evolution are still useful. For example, when Rubin (1995) studied the features of ballads, epic poems, and counting-out rhymes that made for the relatively stable (but not perfect) longevity of information transmitted by oral tradition. Or in the study of the spread and persistence of misinformation (Lewandowsky et al., 2017).

#### Time

There is one important dimension that has been implicit throughout my writing here but is not directly represented in the expanded taxonomies themselves: **time**, which is integral to any form of memory. For example, how do the various systems function across time (e.g., what is their duration), at what points in the past did the systems first emerge (hence my discussion of evolution), and how might the systems or their interactions change in the future (a topic we touched on in Finley et al., 2018)?

There have been a few frameworks even more expansive and ambitious than my own that have included time, which I will briefly review. Henriques (2003) proposed a conceptual framework, the Tree of Knowledge System, that encompasses the evolution of behavioral complexity over the time course of the universe, highlighting the emergence of four qualitatively different dimensions of existence: matter, life, mind, and culture. Gillings et al. (2016) considered the total information in the biosphere of Earth, and outlined transitions in information storage and replication across the evolutionary substrates of chemistry, biology, culture, and technology.

Donald (1991) proposed a broad and detailed theory on the origin of modern human cognition and culture across three evolutionary transitions each marked by emergence of new forms of memory. His theory holds that ancient hominids possessed procedural and episodic memory, then the first transition was mimetics (the ability to mime), followed by spoken language (making narrative culture possible), and finally exograms (written language, or technological external memory, making theoretical culture possible). Kaput and Shaffer (2002) built on Donald's theory and proposed that computational representation has marked a fifth stage of cognition, making virtual culture possible. Along those lines, an edited volume by Ziman (2003) further explored evolutionary perspectives on technological change.

Goonatilake (1991) proposed three lineages of information flow that have stored and transmitted information throughout the history of life on Earth: genetic, neural-cultural (including memory in the brain and social external memory), and exosomatic (technological external memory). Bates (2006) expanded on Goonatilake's framework by proposing information forms that correspond to each lineage, as well as adding a fourth lineage, residue (traces of degrading technological external memory).

#### Entropy

Throughout my writing here, I have touched on the ancient history behind our human memory systems, alluding to the depth of time from which we have all emerged. Memory transmits information across time. In this way, *memory is a countervailing force against entropy*, which is the tendency toward disorder. Life is a kind of organized matter, only a temporary aberration in the arc of the universe toward dissolution, an eddy of order in the tides of chaos. Entropy dissolves order over time. But memory is a force that transcends time. Structures of the past, remembered in the present, influence the future. As self-aware assemblages of matter, we humans are not just children of the universe, but also its eyes and its mind. With all the various forms of memory that comprise us, we endow meaning where otherwise there would be none, for however long we can.

## Footnotes

^1^Some text in the current manuscript is repurposed, with permission, from that work.

^2^I will interchangeably refer to memory in the brain as neurological memory.

^3^“Modal model” is a phrase first coined by Murdock (1967) to describe the consensus among researchers (including a 1965 technical report by Atkinson and Shiffrin, 1965) about a multi-store model of memory. The term “modal” here refers to commonality, as in the statistical mode, and indeed Atkinson and Shiffrin's model has become “one of the most well-known and highly cited theories in the field” (Wixted, 2024). The model also features separate stores for information from different sensory modalities (e.g., visual vs. auditory) across all three structural components, but that term “modality” is unrelated to the phrase “modal model”. Clarifying the origin and meaning of this phrase was prompted by an anonymous reviewer, and resolved in personal communication with R. Atkinson, R. Shiffrin, and K. Malmberg III, November 14–18, 2024.

^4^Operant conditioning does not fit neatly into this taxonomy, as it may or may not involve explicit memory of prior consequences.

^5^In the second expansion to the taxonomy, these definitions will be updated to refer to information stored inside or outside of an individual (i.e., not limited to the brain).

^6^In the third expansion to the taxonomy, this definition will be updated to replace “inanimate” with “human-made” in order to accommodate the addition of natural external memory.

^7^One study estimated 20 billion memory T cells in the skin and 10 billion circulating in the blood (Clark et al., 2006). This is the same order of magnitude as the number of neurons in the brain, with one estimate being 86 billion total with 16 billion in the cerebral cortex (Herculano-Houzel, 2009). However, individual neurons are each part of the networked pattern for many memories, whereas individual memory T and B cells are each specific to one antigen (and thus one memory), so the capacity for immunological memory is much more limited.

^8^The concepts of *culture* and *collective memory* are usually treated as related but distinct, with culture having a broader scope and including present practices and norms while collective memory focuses on the past. However, given my expansive view of memory as transmission of information across time, I will similarly adopt a more expansive view of collective memory that is essentially synonymous with culture. That said, Wang (2008) provides a cogent definition of culture and its relation to collective memory.

^9^Although see Erdelyi (2006) for a broader conception of repression as being akin to inhibition or denial, and not necessarily tied to defense or unconsciousness.

^10^For the interplay of high-tech external memory and *individual* memory, see: Fawns (2011, 2020), Finley et al. (2018), Groes (2016), Marsh and Rajaram (2019), Smart et al. (2017), Storm and Soares (2024), Wang (2022), and Wang and Hoskins (2024).

^11^Note the parallel to the adage, “We shape our tools, and thereafter our tools shape us,” by Culkin (1967) in reference to the ideas of Marshall McLuhan.

^12^The idea of evolution as a learning process is exploited in computer science by the use of genetic algorithms (Holland, 1992), a type of machine learning that simulates natural selection on a population of candidate solutions to an optimization problem.

^13^The overwhelmingly positive student evaluations included these comments: “Wonderful experience to see memory from all different types of disciplines.” and “I thought this class challenged me to think about my use of memory in different ways. I have found myself thinking and talking about the things we talked about in this class with my friends and family outside of class.”
